# Network-based analysis and experimental validation of identified natural compounds from Yinchen Wuling San for acute myeloid leukemia

**DOI:** 10.3389/fphar.2025.1591164

**Published:** 2025-05-30

**Authors:** Biyu Zhang, Denggang Fu, Xin Wang, Xuelei Hu

**Affiliations:** ^1^ Key Laboratory of Green Chemical Engineering Process of Ministry of Education, Hubei Key Laboratory of Novel Reactor and Green Chemical Technology, School of Chemical Engineering and Pharmacy, Wuhan Institute of Technology, Wuhan, China; ^2^ School of Medicine, Jiujiang University, Jiujiang, China; ^3^ Medical University of South Carolina, Charleston, SC, United States

**Keywords:** acute myeloid leukemia, Yinchen Wuling San, network pharmacology, molecular docking, molecular dynamics simulation, pharmacological target

## Abstract

**Objective:**

Traditional Chinese medicine (TCM) has garnered attention for its potential in cancer therapy. Yinchen Wuling San (YWLS), a classical herbal formula, has been traditionally used for liver-related conditions, but its bioactive components and molecular mechanisms relevant to hematologic malignancies such as acute myeloid leukemia (AML) remain unclear. This study aims to identify the active compounds and potential molecular targets of Yinchen Wuling San in the context of AML through network pharmacology analysis, and to experimentally validate the effects of selected candidate compounds in AML models.

**Methods:**

Active ingredients from six YWLS herbs were screened via the TCMSP database using oral bioavailability ≥30% and DL ≥0.18 thresholds. Targets were predicted using SwissTargetPrediction, and AML-related genes were obtained from DisGeNET and GeneCards. Key overlapping targets were analyzed via STRING PPI networks and GO/KEGG enrichment. Molecular docking was performed between three core compounds (genkwanin, isorhamnetin, quercetin) and hub proteins (e.g., SRC) using Sybyl-X. ADME profiles were predicted using SwissADME, and molecular dynamics simulations (GROMACS) assessed complex stability. These compounds were further evaluated *in vitro* (viability, apoptosis, cell cycle, RT-qPCR, flow cytometry) and *in vivo* using an AML xenograft mouse model.

**Results:**

Of 621 YWLS targets, 113 overlapped with 1,247 AML-related genes. PPI analysis identified hub genes, including AKT1, SRC, and EGFR. Enrichment analysis highlighted PI3K-AKT, MAPK, and JAK-STAT pathways. Genkwanin, isorhamnetin, and quercetin were predicted to target SRC, with strong molecular docking affinities. ADME analysis suggested favorable pharmacokinetics, and molecular dynamics simulations confirmed structural stability. *In vitro*, these compounds exhibited dose-dependent cytotoxicity, induced apoptosis, modulated the cell cycle, and downregulated SRC expression. Notably, Genkwanin promoted CD8^+^ T cell proliferation and inhibited leukemia growth, improving survival in a leukemia xenograft model.

**Conclusion:**

YWLS compounds, particularly Genkwanin, exhibit significant anti-leukemic activity via apoptosis induction, cell cycle modulation, and promote T cells proliferation. Genkwanin emerges as a promising therapeutic candidate for AML, warranting further clinical investigation.

## Introduction

Acute myeloid leukemia (AML) is one of the most common and severe types of malignant leukemia in adults and children worldwide (Cai and Levine, 2019). It is an aggressively hematopoietic malignancy that characterized with high heterogeneity, an increased number of accumulative immature clonal myeloid cells in the bone marrow and blood, inhibiting the hematopoiesis of normal blood cells, leading high mortality rate (Bruzzese et al., 2025; Juliusson et al., 2009). Despite advances in oncology, AML remains an aggressive hematopoietic malignancy characterized by rapid progression, marked heterogeneity, and poor clinical outcomes. The 5-year survival of patients with AML has barely improved over 40 years. The severity of AML is reflected in its persistently high mortality rate; the 5-year survival rate has shown only modest improvement over the past 4 decades. According to the Statistics of the American Cancer Society the overall survival rate (OS) of AML patients under 60 years old in recent 5 years is 26% (Miller et al., 2019). The 5-year OS of elderly patients over 60 years old is only 3%–8%, and the median OS is less than 1 year (Pandolfi et al., 2015; Zhang B. et al., 2021; Jing et al., 2024). Currently, the main treatments for AML are chemotherapy, molecular targeted therapy, immune-based therapy, and hematopoietic stem cell transplantation (Yang and Wang, 2018; Chen T. et al., 2024; Fu et al., 2021a). Although these advanced treatments offer available therapeutic options. However, these approaches are limited by issues such as drug resistance, disease relapse, high toxicity, and unsatisfactory long-term outcomes. More than half of young patients face the risk of relapse or refractory treatment, and the elderly or debilitated patients are unable to tolerate the serious side effects of conventional chemotherapy, 40% of patients with traditional regimen still fail to achieve complete response and have low OS (O'Donnell et al., 2017). High relapse was observed in patients with chemotherapy with the reformulation of standard therapy, such as Vyxeo™, cytarabine and daunorubicin liposome administration (Kantarjian et al., 2021). Novel FLT3 inhibitor is restricted to be applied for mutation targeted therapy (Sanz et al., 2009). Antibody-toxin Gemtuzumab ozogamicin conjugates that targeting CD33^+^ AML with high toxicity (Venugopal et al., 2021). Although chimeric antigen receptor (CAR) T-cell therapy offers a novel immunotherapy approach, its clinical application is hampered by high costs and risks of serious adverse effects, such as cytokine release syndrome (Tabata et al., 2021). These significant limitations underline the urgent need for novel, safer, and more effective therapeutic strategies for AML.

Given these challenges, increasing attention has turned to alternative and complementary medical approaches. Traditional Chinese Medicine (TCM), with its holistic treatment philosophy and multi-targeted therapeutic properties, has shown promise in supporting cancer therapy, including hematologic malignancies. Several TCM-derived phytochemicals, such as curcumin, berberine, and resveratrol, have been found to enhance chemotherapy efficacy and sensitize AML cells to treatment (Jin et al., 2013; Xiang et al., 2019). However, these effects are attributed to individual compounds rather than TCM as a comprehensive therapeutic system. Some clinical studies have reported that certain Chinese herbal medicines or integrative approaches combining herbal medicine with conventional chemotherapy may improve complete response rates and help mitigate chemotherapy-induced adverse effects such as diarrhea, vomiting, nausea, and bleeding or infection resulting from bone marrow suppression (Fleischer et al., 2017; Chen and Zhuang, 2024).

Yinchen Wuling San Prescription (YWLS), a classical TCM prescription originating from Synopsis of Golden Chamber, consists of six herbals: *Artemisiae Scopariae Herba* (ASH, Chinese name: Yinchen), *Poria Cocos* (PC, Chinese name: Fuling), *Alisma Orientale* (AO, Chinese name: Zexie), *Atractylodes Macrocephala Koidz* (AMK, Chinese name: Baizhu), *Polyporus Umbellatus* (PU, Chinese name: Zhuling), and *Cinnamomi Ramulus* (CR, Chinese name: Guizhi) (Haiqiang et al., 2016). Traditionally, YWLS is prepared by grinding the herbs into fine powder and taken orally with rice soup on an empty stomach, three times daily, as recorded in the ancient medical text Jin Gui Yao Lue. In modern clinical settings, YWLS is commonly administered as a decoction. The raw herbs are soaked and boiled twice (first for 2 h, then for 1 h), with the filtrates combined and concentrated into a specific volume. The extract can then be dried into granules or processed into capsules for ease of use (Leong et al., 2024). For experimental use, the decoction is typically concentrated and administered orally in animal models or reconstituted in suitable solvents for *in vitro* studies (Wu et al., 2022). YWLS has been widely used in the treatment of liver-related disorders such as icteric hepatitis (Zhang Y. et al., 2021), hepatic fibrosis, and non-alcoholic fatty liver disease (Liu and Zhao, 2024). Several individual herbal components of YWLS have shown potential anti-leukemic activities. For instance, Alisol B 23-acetate (ABA), a bioactive compound isolated from Alismatis Rhizoma (AO), exhibited P-glycoprotein (P-gp) reversal activity in a multidrug-resistant (MDR) leukemia cell line (K562-DR) (Wang et al., 2004), and demonstrated synergistic effects when combined with chemotherapeutic agents such as cisplatin and fluorouracil (5-FU) (Fong et al., 2007). In a study by Willimott et al., the water extract of Alismatis Rhizoma (AO), one of the herbal components of the traditional formula Long Dan Xie Gan Wan, was found to exhibit cytotoxic activity against human promyelocytic leukemia HL-60 cells, suggesting that AO extract may possess anti-leukemic properties when used as part of multi-herbal preparations (Willimott et al., 2009). Triterpenes separated from PC were identified to play a key role in anti-leukemia (Xu et al., 2020; Choi, 2015; Ukiya et al., 2002). *AMK* can induce apoptosis of human leukemia cells through increased production of reactive oxygen species (Huang et al., 2005; Huang et al., 2016). Polyporusterone derived from PU exhibited cytotoxic action on leukemia cell proliferation (Ohsawa et al., 1992). *CR* can alleviate chemoradiotherapy-induced gastrointestinal reaction, such as nausea, vomiting and constipation (Bu et al., 2024). Extracts isolated from ASH have anti-inflammation and anti-cancer activities (Cai et al., 2020). For example, Capillin potently inhibited human leukemia HL-60 cells growth through inducing apoptosis (Masuda et al., 2015). These indicated that active ingredients from YWLS may exert synergistic anti-leukemia effects. Despite these promising observations, the bioactive components of YWLS and their anti-leukemic mechanisms remain largely unexplored. Comprehensive elucidation of its molecular mechanisms is crucial for potential development into evidence-based, adjunctive AML therapies.

Network pharmacology acting as an emerging analytical approach has been widely employed to predict potential molecular mechanisms of pharmacological action for TCM through integrating drugs, diseases, and their corresponding targets into biomolecular network (Li and Zhang, 2013; Sun et al., 2021). In this study, we utilized a network pharmacology approach to systematically predict the active compounds, targets, and pathways associated with YWLS against AML (Supplementary Figure S1). Molecular docking and ADME (Absorption, Distribution, Metabolism, and Excretion) analysis were employed to evaluate drug-target interactions and drug-likeness. Molecular dynamics simulations were conducted to assess the stability of compound-protein complexes. Finally, the anti-leukemic potential of selected active ingredients was validated through *in vitro* assays. This study aims to uncover the underlying pharmacological mechanisms of YWLS against AML and to provide a scientific basis for its potential application as a novel anti-AML therapeutic strategy. Further *in vivo* investigations will be warranted to strengthen these findings.

## Methods and materials

### Identification of active ingredients of YWLS and its pharmacological targets

YWLS, a common traditional Chinese prescription, contains six herbs ASH, PC, AO, AMK, CR, and PU. A variety of compounds identified from these herbs were found to have anti-inflammation/oxidant/tumor activities and immune regulatory functions (Huang et al., 2016). The active ingredients of these herbs were retrieved from TCMSP database (https://tcmsp-e.com/tcmsp.php) (Ru et al., 2014) with parameters of drug-like properties (DL) ≥0.18 and oral bioavailability (OB) ≥30%.

The pharmacological targets of the ingredients of YWLS were screened from SwissTargetPrediction database (http://www.swisstargetprediction.ch/) (Daina et al., 2019). Additional unpredicted targets for these ingredients were included through literature review. The UniProt database (https://www.drugbank.ca/) (Toth et al., 2020) was employed to standardize gene names of targets. While this approach is widely adopted in network pharmacology studies, it may be biased toward well-characterized and frequently studied targets due to the nature of the underlying training data in these databases.

### Screening disease targets

Pathological targets related to acute myeloid leukemia (AML) were obtained from the DisGeNET database (https://www.disgenet.org/) with a gene-disease association (GDA) score ≥0.1 and the GeneCards database (https://www.genecards.org/) with a relevance score ≥10. After removing duplicate genes, the intersecting targets of YWLS active ingredients and AML-related genes were identified using the “VennDiagram” package (version 1.7.3) in R software (version 4.2.0).

### Protein-protein interaction (PPI) network and gene enrichment analysis

The STRING database (https://string-db.org/, version 11.5) was used to analyze interactions among the intersected genes, with a minimum interaction score threshold set at 0.4 (Szklarczyk et al., 2021). The resulting PPI network was visualized using Cytoscape (version 3.8.0) (Shannon et al., 2003). Densely connected modules were identified using the MCODE plugin (Bader and Hogue, 2003) with default parameters (Degree Cutoff = 2, Node Score Cutoff = 0.2, K-Core = 2, Max Depth = 100).

Network centrality was assessed using the CytoNCA plugin (Tang et al., 2015) (based on betweenness, closeness, and degree centralities), and hub genes were further screened with the cytoHubba plugin (Chin et al., 2014) using the Maximal Clique Centrality (MCC) method.

Gene Ontology (GO) functional enrichment (including biological process [BP], molecular function [MF], and cellular component [CC]) and Kyoto Encyclopedia of Genes and Genomes (KEGG) pathway analyses were conducted using the “clusterProfiler” R package (version 4.2.2) with a cutoff of *p*. adjust <0.05. Key AML-related signaling pathways were visualized using the Pathview R package (Luo and Brouwer, 2013) (version 1.38.0).

### Construction of drug-target-disease network

Relationships between active ingredients, targets, and disease were organized using Perl scripts (Perl version 5.32.1). The integrated Drug-Target-Disease network was visualized by Cytoscape (version 3.8.0) (Shannon et al., 2003).

### Gene expression profiling and prognostic evaluation of identified targets in AML

Gene expression data (log2(FPKM+1) format) and clinical information for AML patients were downloaded from the UCSC Xena database (https://xenabrowser.net/datapages/). Wilcoxon rank-sum test was used to compare gene expression levels between normal and AML samples. Univariate Cox regression analysis was performed using the “survival” R package (version 3.4.0) to identify targets associated with overall survival (OS). A *p*-value <0.05 was considered statistically significant.

### Molecular docking

Structures of active compounds were downloaded from the TCMSP database (https://tcmsp-e.com/tcmsp.php) in.mol2 format, and the crystal structures of target proteins were retrieved from the RCSB Protein Data Bank (https://www.rcsb.org/). Molecular docking was performed using Sybyl-X 2.0 software. Ligand Preparation: Small molecules underwent energy minimization using the Tripos force field, applying 1,000 iterations and a convergence criterion of 0.005 kcal/(mol·Å). Protein Preparation: Proteins were preprocessed by removing water molecules, adding hydrogens, and optimizing side chains. Docking scores were calculated, and binding poses were evaluated. Docking interactions were visualized using PyMOL software (version 2.5.4).

### ADME analysis

The pharmacokinetic properties (absorption, distribution, metabolism, and excretion), drug-likeness, and medicinal chemistry profiles of the selected compounds were evaluated using the SwissADME database (http://www.swissadme.ch/) (Daina et al., 2017).

### Molecular dynamics simulation

Molecular dynamics simulations were conducted using GROMACS software (version 2020.3) (Abraham et al., 2015; Van Der Spoel et al., 2005). Ligand Topology: Ligand parameters were generated via SwissParam (https://www.swissparam.ch/) (Zoete et al., 2011). Force Field and Solvation: CHARMM36 force field and the TIP3P explicit water model were applied (Kutzner et al., 2015; Ul Haq et al., 2017). Complexes were solvated in a cubic box with at least 1.0 nm distance from the box edges, neutralized with Na+/Cl− ions. Energy Minimization: Conducted using 50,000 steps each of steepest descent and conjugate gradient methods. System Equilibration: NVT ensemble (constant number of particles, volume, and temperature) for 100 ps at 300 K using the V-rescale thermostat. NPT ensemble (constant number of particles, pressure, and temperature) for 100 ps at 1 atm using the Parrinello-Rahman barostat (Fatima et al., 2014). Production Run: 50 ns MD simulation with a 2 fs timestep using the leap-frog integration algorithm. Trajectory analyses included: Root-Mean-Square Deviation (RMSD) to monitor structural stability. Root-Mean-Square Fluctuation (RMSF) to assess residue flexibility. Radius of Gyration (Rg) to evaluate protein compactness. Solvent Accessible Surface Area (SASA) to explore solvation effects. Hydrogen Bond (Hbond) Analysis to characterize protein-ligand interactions. All analyses were conducted using GROMACS built-in modules and visualized with OriginPro 2022b. Binding Free Energy Calculation: The Molecular Mechanics Poisson-Boltzmann Surface Area (MM-PBSA) method was applied to estimate binding free energies of the protein-ligand complexes using the “g_mmpbsa” package. The total binding free energy was calculated based on the last 10 ns of the simulation trajectory (Monteiro et al., 2018; Patidar et al., 2019).

### Cell culture

MOLM14 cells were cultured in RPMI1640 medium supplemented with 10% fetal bovine serum and 1% penicillin/strptomycin and grown at 37°C with 5% CO_2_.

Quercetin, Isorhamnetin, and Genkwanin (Sigma-Aldrich) were dissolved in Dimethylsulfoxide (DMSO) to prepare the stock solutions of 100 mmol/L. Different Genkwanin, Quercetin, and Isorhamnetin working solutions of 10, 20, 40, and 80 μmol/L were diluted from the stock solutions and used to treat MOLM14 cells at different assays.

### Cell viability analysis

MOLM14 cells (1 × 10^5^) were plated in 96-well plates. Cells per well were treated for 72 h with various concentrations of different compounds followed by Viability Dye eFluor™ 506 (Invitrogen, catalog: 65-0866-14) to each well for 30 min incubation. The cell viability was then assessed by flow cytometry. The cell viability was determined by normalizing to vehicle group.

### Dose-response modeling and interpolation

To estimate tumor cell growth inhibition at additional concentrations, a four-parameter logistic (4 PL) model was fitted to the experimental dose-response data for Quercetin, Isorhamnetin, and Genkwanin. The fitted model was used to interpolate or extrapolate inhibition rates at untested concentrations of 0.1, 1, 10, and 100 μM. The 4 PL model is defined as:

$$Y=\text{Bottom}+\left(\text{Top }‐\text{ Bottom}\right)\;/\;\left[1+{\left(X\;/\;\text{IC}50\right)}^{H}\right]$$

where Y is the predicted inhibition rate, X is the drug concentration, and H is the Hill slope. Curve fitting and prediction were performed using the scipy.optimize.curve_fit function in Python.

### Cell cycle and apoptosis assay

For cell cycle analysis of MOLM14 cells treated with Genkwanin, Quercetin, and Isorhamnetin (10, 20, 40, and 80 μmol/L) for 72-h. Cells in 96-well plate were harvested and washed with 1x phosphate buffered saline (PBS). The washed cells were fixed using Foxp3/Transcription Factor Staining Buffer Set and the Fixation/Permeabilization Kit (Cat.No: 00-5523-00, Invitrogen) for 30 min at room temperature. Ki-67 at indicated concentration was added to each well and incubated at 4°C for 30 min. DAPI was added at 2 mM/L for each well and analyzed on a BD LSRFortessa in 30 min.

Apoptosis assay was performed in MOLM14 cells treated with Genkwanin, Quercetin, and Isorhamnetin (10, 20, 40, and 80 μM) at 72-h following the PE-Annexin V Apoptosis Detection Kit (BD Pharmingen™, catalog: 559763) instructions, and analyzed on a BD LSRFortessa.

### Mitochondria function assay

MOLM14 cells were treated with genkwanin, quercetin, or isorhamnetin (10, 20, 40, and 80 μM) in a 96-well plate for 72 h. After treatment, cells were centrifuged to collect a cell pellet and the supernatant was aspirated. Cells were gently resuspended in pre-warmed (37°C) RPMI 1640 medium containing MitoTracker^®^ Deep Red FM (Thermo Fisher, Cat. No. M22426) and MitoTracker^®^ Red CMXRos (Thermo Fisher, Cat. No. M7512) and incubated for 30 min at 37°C. After staining, cells were washed, resuspended in pre-warmed 1xPBS buffer, and analyzed by flow cytometry. Debris and apoptotic bodies were excluded based on FSC/SSC gating to focus the analysis on intact cells. All samples were prepared and stained under identical conditions, and comparisons were made relative to control-treated cells.

### Quantitative RT-PCR (qRT-PCR)

Total RNA from MOLM14 cells was extracted using the RNeasy Mini Kit (QIAGEN, Cat. No. 74104) according to the manufacturer’s protocol. RNA concentration and purity (A260/A280 ratio) were assessed using a NanoDrop spectrophotometer, and samples with acceptable purity (A260/A280 between 1.8 and 2.0) were used for downstream analysis. One microgram of RNA was reverse transcribed to cDNA using the iScript™ cDNA synthesis kit (Bio-Rad, Cat. No. 1708891). Fifty nanograms of cDNA were used for qPCR on a CFX96 Real-Time PCR Detection System (Bio-Rad). qPCR reactions were performed using SsoAdvanced Universal SYBR Green Supermix (Bio-Rad, Cat. No. 172-5270) with the following program: 95°C for 30 s; 40 cycles of 95°C for 15 s and 60°C for 1 min.

Relative gene expression was calculated using the 2^−ΔΔCT^ method and normalized to GAPDH expression. Primers for SRC gene expression were described previously (Zhang et al., 2020). The 72-h treatment time point was selected to capture late-stage transcriptional changes, including those involved in apoptotic and stress response pathways. Despite partial loss of viability at this time point, sufficient viable cells remained for reliable RNA extraction and analysis.

### Flow cytometry

All antibodies used for intracellular, or surface staining used in this study were purchased from eBioscience (San Diego, CA) or BD Bioscience (San Jose, CA) or Biolegend (San Diego, CA) unless otherwise stated. For cell surface antibody staining, each antibody was added at recommended concentration and incubated at 4°C for 30 min by blocking with 20% FBS for 15 min at 4°C, which prevents non-specific binding. For intracellular and cytokines staining, cells were fixed with Foxp3/Transcription Factor Staining Buffer Set and the Fixation/Permeabilization Kit (Cat. No: 00-5523-00, Invitrogen) for 30 min at room temperature followed by permeabilization with 1x permeabilization buffer. For the cytokine staining, samples were stimulated with phorbol 12-myristate 13-acetate (PMA) (50 ng/mL; Sigma-Aldrich), ionomycin (1 μg/mL; Sigma-Aldrich), and in the presence of GolgiStop monensin (1 μg/mL) for 4–5 h at 37°C. Cells were washed twice with 1X permeabilization buffer. Finally, samples were run on BD LSRII, BD LSR Fortessa (X-20) and analyzed using FlowJo 10.7.0 software (TreeStar).

### T cell proliferation assay

Splenocytes were harvested from C57BL/6 mice and purified using a Pan T Cell Isolation Kit (Catalog No. 130-095-130). T cells were labeled with CFSE (5 μM) for 5 min at room temperature. A total of 1 × 10^5^ cells per well were seeded into a 96-well round-bottom plate containing 200 μL of RPMI 1640 medium supplemented with 10% FBS, 2.5% 1M HEPES, 1% non-essential amino acids, 1% penicillin/streptomycin, and 1% sodium pyruvate.

Quercetin, Isorhamnetin, and Genkwanin were added to the respective wells at final concentrations of 0, 10, 20, 40, and 80 μM, with 0.1% DMSO serving as the vehicle control. After 72 h of culture, cells were harvested and analyzed by flow cytometry. Staining included CD4 (PE), CD8 (PE-Tex-Red), and viability dye (eFluor™ 506) to assess cell proliferation.

#### *In vivo* study of Genkwanin using a leukemia xenograft model

To evaluate the anti-leukemic efficacy of Genkwanin *in vivo*, we developed a human leukemia xenograft model using 8–10-week-old NOD. Cg-Prkdc^scid^ Il2rg^tm1Wjl^/SzJ (NSG) mice, both male and female. Each mouse was injected intravenously with 1 × 10^5^ MOLM14^gfp^ cells. Four days after leukemia cell engraftment, mice were randomized into treatment and control groups. The treatment group received intraperitoneal injections of Genkwanin (5 mg/kg) twice weekly for 3 weeks, while the control group received vehicle only.

To simulate and evaluate the potential immunomodulatory effects of Genkwanin within a limited human immune context, mice received weekly intravenous adoptive transfers of 1 × 10^6^ human peripheral blood mononuclear cells (PBMCs) isolated from healthy donors, starting on the first day of treatment. This approach enabled partial and transient reconstitution of human immune cells—primarily T lymphocytes—allowing for exploratory assessment of T cell-mediated responses, particularly CD8^+^ T cell activity, in the presence of Genkwanin.

Several studies have adopted similar strategies, using NSG mice humanized with PBMCs with or without leukemic cell co-engraftment to explore immunomodulatory functions of therapeutic agents or gene targets in leukemia and lymphoma models (Fu et al., 2021b; Adom et al., 2020; Wellhausen et al., 2023; Pyzer et al., 2017). These models provide valuable insight into human immune cell dynamics and treatment responses, despite not fully recapitulating the patient-matched immune milieu of autologous PDX systems.

Survival was monitored daily, and on Day 21 post-transfer, tumor burden and frequencies of CD8^+^ T cells in the bone marrow were quantified by flow cytometry. All animal experiments were conducted in accordance with protocols approved by the Animal Ethics Committee at Jiujiang University (Approval No. JJU20240076).

## Results

### Uncovering active ingredients in YWLS and their target genes

A total of 58 active ingredients in YWLS were identified through the TCMSP database and supplemented with findings from literature review (Table 1). These include 13 ingredients from ASH, seven from CR, 10 from AO, 11 from PU, 15 from PC, and seven from AMK. Notably, several compounds are shared between different sources: ASH and CR both contain beta-sitosterol; PC and PU share ergosta-7,22E-dien-3beta-ol and cerevisterol; AO and CR both include sitosterol; and PU and CR share peroxyergosterol. To identify the targets of these active ingredients, 621 targets were retrieved from the Swiss Target Prediction database (Supplementary Table S1).

**TABLE 1:** Active components of YWLS were selected based on OB and DL parameters.

Mol id	Molecule name	OB (%)	DL	Herb name
MOL000359	Sitosterol	36.91	0.75	Alisma Orientale
MOL000830	Alisol B	34.47	0.82	Alisma Orientale
MOL000831	Alisol B monoacetate	35.58	0.81	Alisma Orientale
MOL000832	alisol,b,23-acetate	32.52	0.82	Alisma Orientale
MOL000849	16β-methoxyalisol B monoacetate	32.43	0.77	Alisma Orientale
MOL000853	alisol B	36.76	0.82	Alisma Orientale
MOL000854	alisol C	32.7	0.82	Alisma Orientale
MOL000856	alisol C monoacetate	33.06	0.83	Alisma Orientale
MOL002464	1-Monolinolein	37.18	0.3	Alisma Orientale
MOL000862	[(1S,3R)-1-[(2R)-3,3-dimethyloxiran-2-yl]-3-[(5R,8S,9S,10S,11S,14R)-11-hydroxy-4,4,8,10,14-pentamethyl-3-oxo-1,2,5,6,7,9,11,12,15,16-decahydrocyclopenta [a]phenanthren-17-yl]butyl] acetate	35.58	0.81	Alisma Orientale
MOL000279	Cerevisterol	37.96	0.77	Polyporus Umbellatus
MOL000282	ergosta-7,22E-dien-3beta-ol	43.51	0.72	Polyporus Umbellatus
MOL000796	(22e,24r)-ergosta-6-en-3beta,5alpha,6beta-triol	30.2	0.76	Polyporus Umbellatus
MOL000797	(22e,24r)-ergosta-7,22-dien-3-one	44.88	0.72	Polyporus Umbellatus
MOL000798	ergosta-7,22-diene-3β-ol	43.51	0.72	Polyporus Umbellatus
MOL000801	5alpha,8alpha-epidioxy-(22e,24r)-ergosta-6,22-dien-3beta-ol	44.39	0.82	Polyporus Umbellatus
MOL011169	Peroxyergosterol	44.39	0.82	Polyporus Umbellatus
MOL000816	ergosta-7,22-dien-3-one	44.88	0.72	Polyporus Umbellatus
MOL000817	ergosta-5,7,22-trien-3-ol	46.18	0.72	Polyporus Umbellatus
MOL000820	Polyporusterone E	45.71	0.85	Polyporus Umbellatus
MOL000822	Polyporusterone G	33.43	0.81	Polyporus Umbellatus
MOL000273	(2R)-2-[(3S,5R,10S,13R,14R,16R,17R)-3,16-dihydroxy-4,4,10,13,14-pentamethyl-2,3,5,6,12,15,16,17-octahydro-1H-cyclopenta [a]phenanthren-17-yl]-6-methylhept-5-enoic acid	30.93	0.81	Poria Cocos
MOL000275	Trametenolic acid	38.71	0.8	Poria Cocos
MOL000276	7,9 (11)-dehydropachymic acid	35.11	0.81	Poria Cocos
MOL000279	Cerevisterol	37.96	0.77	Poria Cocos
MOL000280	(2R)-2-[(3S,5R,10S,13R,14R,16R,17R)-3,16-dihydroxy-4,4,10,13,14-pentamethyl-2,3,5,6,12,15,16,17-octahydro-1H-cyclopenta [a]phenanthren-17-yl]-5-isopropyl-hex-5-enoic acid	31.07	0.82	Poria Cocos
MOL000282	ergosta-7,22E-dien-3beta-ol	43.51	0.72	Poria Cocos
MOL000283	Ergosterol peroxide	40.36	0.81	Poria Cocos
MOL000285	(2R)-2-[(5R,10S,13R,14R,16R,17R)-16-hydroxy-3-keto-4,4,10,13,14-pentamethyl-1,2,5,6,12,15,16,17-octahydrocyclopenta [a]phenanthren-17-yl]-5-isopropyl-hex-5-enoic acid	38.26	0.82	Poria Cocos
MOL000287	3beta-Hydroxy-24-methylene-8-lanostene-21-oic acid	38.7	0.81	Poria Cocos
MOL000289	Pachymic acid	33.63	0.81	Poria Cocos
MOL000290	Poricoic acid A	30.61	0.76	Poria Cocos
MOL000291	Poricoic acid B	30.52	0.75	Poria Cocos
MOL000292	Poricoic acid C	38.15	0.75	Poria Cocos
MOL000296	Hederagenin	36.91	0.75	Poria Cocos
MOL000300	Dehydroeburicoic acid	44.17	0.83	Poria Cocos
MOL000072	8β-ethoxy atractylenolide Ⅲ	35.95	0.21	Atractylodes Macrocephala Koidz
MOL000033	(3S,8S,9S,10R,13R,14S,17R)-10,13-dimethyl-17-[(2R,5S)-5-propan-2-yloctan-2-yl]-2,3,4,7,8,9,11,12,14,15,16,17-dodecahydro-1H-cyclopenta [a]phenanthren-3-ol	36.23	0.78	Atractylodes Macrocephala Koidz
MOL000028	α-Amyrin	39.51	0.76	Atractylodes Macrocephala Koidz
MOL000049	3β-acetoxyatractylone	54.07	0.22	Atractylodes Macrocephala Koidz
MOL000021	14-acetyl-12-senecioyl-2E,8E,10E-atractylentriol	60.31	0.31	Atractylodes Macrocephala Koidz
MOL000020	12-senecioyl-2E,8E,10E-atractylentriol	62.4	0.22	Atractylodes Macrocephala Koidz
MOL000022	14-acetyl-12-senecioyl-2E,8Z,10E-atractylentriol	63.37	0.3	Atractylodes Macrocephala Koidz
MOL001736	(−)-taxifolin	60.51	0.27	Cinnamomi Ramulus
MOL000358	Beta-sitosterol	36.91	0.75	Cinnamomi Ramulus
MOL000359	Sitosterol	36.91	0.75	Cinnamomi Ramulus
MOL000492	(+)-catechin	54.83	0.24	Cinnamomi Ramulus
MOL000073	ent-Epicatechin	48.96	0.24	Cinnamomi Ramulus
MOL004576	Taxifolin	57.84	0.27	Cinnamomi Ramulus
MOL011169	Peroxyergosterol	44.39	0.82	Cinnamomi Ramulus
MOL000354	Isorhamnetin	49.6	0.31	Artemisiae Scopariae Herba
MOL000358	Beta-sitosterol	36.91	0.75	Artemisiae Scopariae Herba
MOL004609	Areapillin	48.96	0.41	Artemisiae Scopariae Herba
MOL005573	Genkwanin	37.13	0.24	Artemisiae Scopariae Herba
MOL007274	Skrofulein	30.35	0.3	Artemisiae Scopariae Herba
MOL008039	Isoarcapillin	57.4	0.41	Artemisiae Scopariae Herba
MOL008040	Eupalitin	46.11	0.33	Artemisiae Scopariae Herba
MOL008041	Eupatolitin	42.55	0.37	Artemisiae Scopariae Herba
MOL008043	Capillarisin	57.56	0.31	Artemisiae Scopariae Herba
MOL008045	4′-Methylcapillarisin	72.18	0.35	Artemisiae Scopariae Herba
MOL008046	Demethoxycapillarisin	52.33	0.25	Artemisiae Scopariae Herba
MOL008047	Artepillin A	68.32	0.24	Artemisiae Scopariae Herba
MOL000098	Quercetin	46.43	0.28	Artemisiae Scopariae Herba

### Active ingredient-target-disease network analysis

**FIGURE 1:**
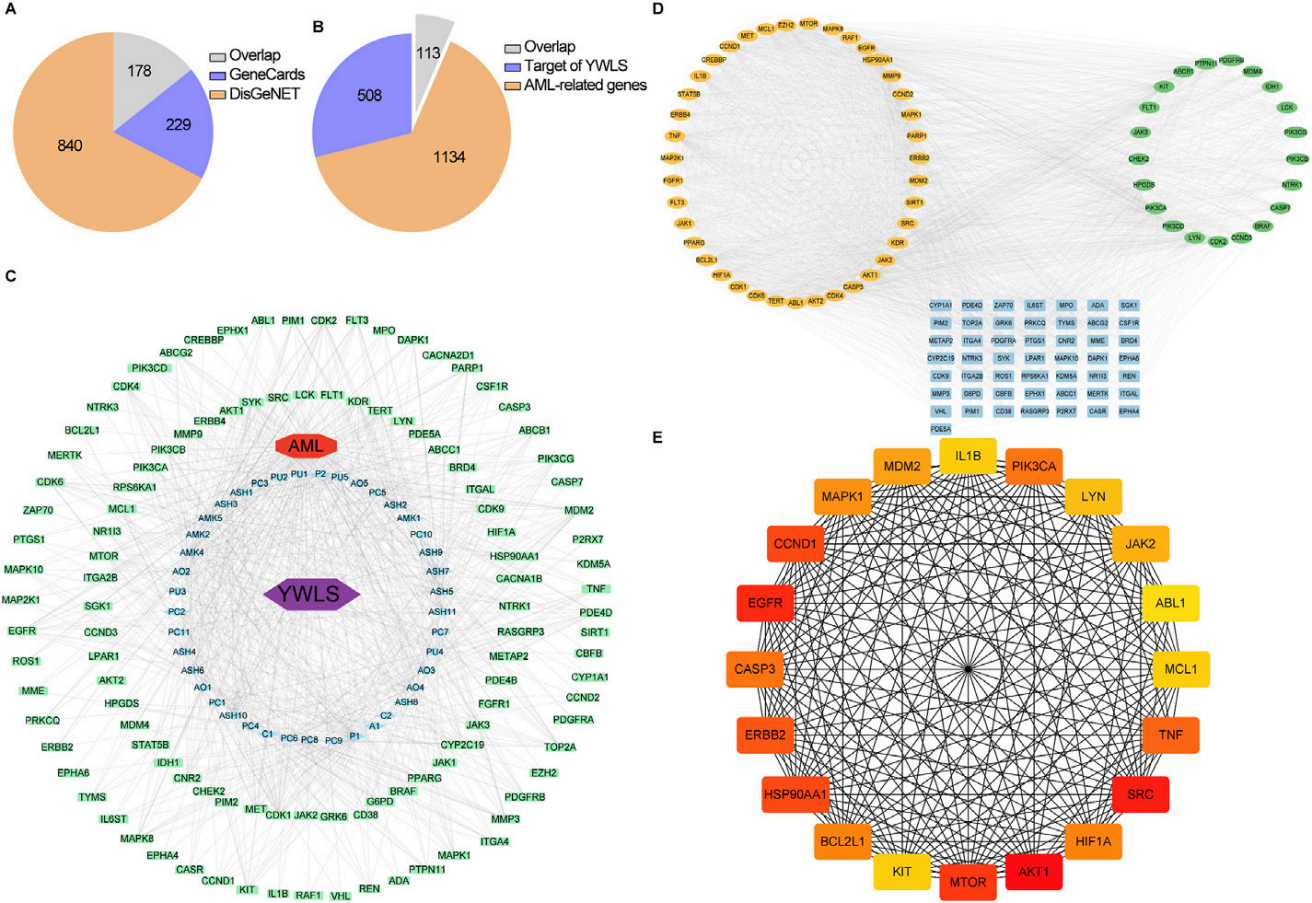
Construction of a compound-target-disease network based on the targets of active ingredients from YWLS and AML-associated genes. **(A)** Screening of acute myeloid leukemia related genes from DisGeNET and GeneCards databases. **(B)** The intersection of targets of active ingredients of YWLS and AML-related genes. **(C)** The active component-target-disease network. The red diamond represented the disease; The light green rectangle represented intersecting targets; The light purple diamond represented YWLS; The blue triangle represented active compounds. The edges represented the connection among active components, targets, and disease. **(D)** Two modules (AKT1 and PIK3CA) identified from the whole PPI network of 113 targets of active ingredients in YWLS and AML-associated genes. **(E)** 20 core targets determined by the degrees. Color represented the target degree.

To identify genes that are potentially related to AML pathogenesis, we retrieved 1,018 and 407 AML disease related genes (ADRGs) from DisGeNET and GeneCards, respectively (Figure 1A). A total of 1,247 ADRGs were obtained after deduplication (Supplementary Table S2). Targets of YWLS and AML were intersected using Venny, 113 targets of the active ingredients from YWLS overlapped with ADRGs (Figure 1B; Supplementary Table S3). Furthermore, we developed YWLS-ingredients-targets-AML network which includes 156 nodes and 841 edges using Perl language and visualized through Cytoscape (Figure 1C). The 14-acetyl-12-senecioyl-2E,8E,10E-atractylentriol, Cerevisterol and 12-senecioyl-2E,8E,10E-atractylentriolareare are active ingredients that have the most targets than other ingredients.

### Protein-protein interaction and gene enrichment analysis of YWLS-AML targets

The PPI network analysis was conducted to explore potential interactions of 113 intersection targets using the STRING database. Two modules were identified through the MCODE plugin via the Cytohubba plugin (Figure 1D). The first module, named AKT1, contains 39 nodes and 1,072 edges, while the second module includes 21 nodes and 168 edges. Among the hub genes, 17 out of 20 genes are part of the first module, including AKT1, SRC, EGFR, MTOR, CCND1, and HSP90AA1. The second module retains PIK3CA, CYN, and KIT as its top genes. Numerous reports have highlighted the association of these genes with AML pathogenesis and inflammation.

Constitutive activation of the PI3K/Akt/mTOR pathway occurs in 50%–80% of AML patients and is linked to reduced OS (Bertacchini et al., 2014; Darici et al., 2020). Downregulation of GLUT3 via the EGFR signaling pathway promotes apoptosis of AML cells (Zhuang et al., 2018). The deregulated activity of Src tyrosine kinases induces malignant transformation, and patients with leukemia have benefited from Src tyrosine kinase inhibitors (Rivera-Torres and San José, 2019). SRC is expressed and activated in most AML primary samples and cell lines, and its inhibitors can suppress the proliferation of AML cell lines and promote apoptosis (MacDonald et al., 2018). Additionally, mutations in CCND1 have been identified in adult AML patients, suggesting its potential as a therapeutic target (Eisfeld et al., 2017).

We also identified 20 hub genes in the network by ranking degree using the Cytohubba plugin in Cytoscape (Figure 1E; Supplementary Table S4). These findings prompted us to conduct a comprehensive analysis of their biological processes through gene function enrichment. Our analysis revealed that these genes are primarily involved in processes such as peptidyl-tyrosine phosphorylation, protein serine/threonine kinase activity regulation, and phosphatidylinositol 3-kinase signaling. The cellular components were predominantly enriched in processes related to transferring phosphorus-containing groups and membrane rafts. The primary molecular functions included protein tyrosine/serine/threonine kinase activity, phosphatase and growth factor binding, and transmembrane receptor protein kinase activity (Supplementary Figure S2A). This suggests that the active ingredients of YWLS may inhibit leukemia growth by regulating kinase activity, protein and growth factor binding, and membrane raft dynamics.

KEGG analysis revealed notably enriched pathways, including PI3K-AKT, MAPK, Ras, JAK-STAT, acute myeloid leukemia, EGFR tyrosine kinase inhibitors, and endocrine resistance (Supplementary Figure S2B). These leukemia-promoting signaling pathways further emphasize the crucial role of these targets in AML pathogenesis and treatment response. Additionally, we observed the involvement of PD-L1 expression, and the PD-1 checkpoint pathway in cancer, indicating that the identified targets may play a role in immunotherapy-based treatment responses by modulating the tumor immune microenvironment.

### Correlation of intersected targets expression with patients’ overall survival

**FIGURE 2:**
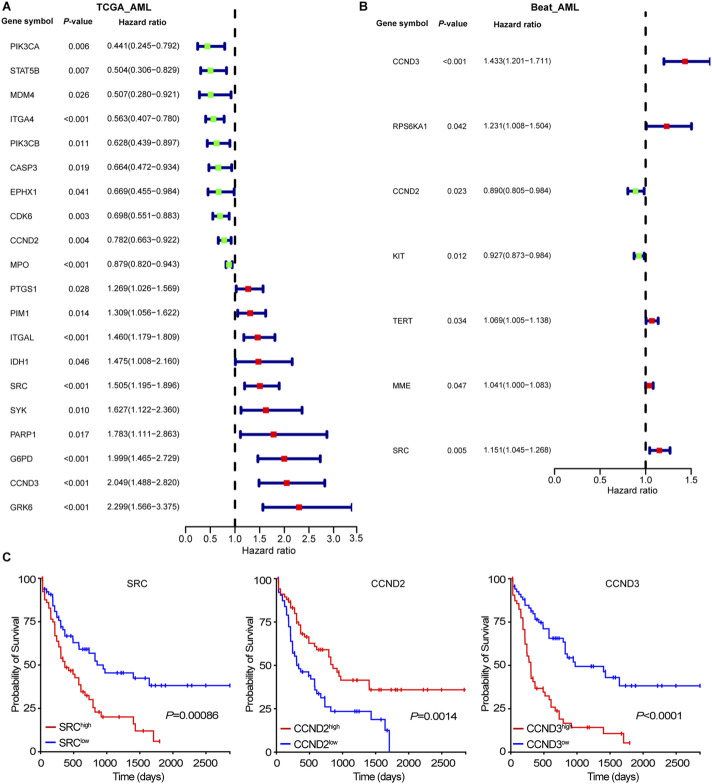
Associations of intersecting targets with patients’ outcome. **(A)** Forest plot represented the associations of intersecting targets with patients’ outcome in TCGA-AML cohort. **(B)** Forest plot represented the associations of intersecting targets with patients’ outcome in Beat-AML cohort. **(C)** The Kaplan-Meier curve of SRC, CCND2, and CCND3 in TCGA-AML cohort.

We explored the relationship between 113 intersection targets and AML prognosis using clinical data from 132 AML patients in the TCGA database. Kaplan-Meier analysis and the log-rank test were applied for survival analysis. Univariate Cox regression identified 20 targets significantly associated with AML prognosis (Figure 2A). Overexpression of PIK3CA, STAT5B, MDM4, ITGA4, PI3CB, CASP3, EPHX1, CDK6, CCND2, and MPO were linked to a favorable prognosis, while higher expression of PTGS1, PIM1, ITGAL, IDH1, SRC, SYK, PARP1, G6PD, CCND3, and GRK6 indicated poor prognosis. Validation using the Beat-AML dataset confirmed the prognostic relevance of CCND2, CCND3, and SRC, aligning with previous studies (Figure 2B). As shown in Figure 2C, low CCND2 and high SRC expression were associated with worse survival outcomes, consistent with earlier reports (Zhang X. et al., 2022; Patel et al., 2019).

### Molecular docking

To explore the potential interactions between bioactive compounds in YWLS and key molecular targets associated with AML, molecular docking studies were performed using SybylX-2.0. We selected three core AML-related proteins—PIK3CA, SRC, and CASP3—based on their relevance in leukemogenesis and their identification as key nodes in our network pharmacology analysis.

Docking simulations revealed that multiple compounds exhibited favorable binding affinities with these proteins. Specifically, five compounds demonstrated excellent affinity (docking score >7), 10 compounds showed good affinity (score 5–7), and six compounds exhibited moderate affinity (score 4–5) (Table 2). These findings may suggest the potential for YWLS-derived compounds to modulate AML-related signaling pathways through direct interaction with critical molecular targets.

**TABLE 2:** Binding affinities between key target genes (SRC, PIK3CA, CASP3) and active YWLS compounds by molecular docking.

Target	PDB id	Mol ID	Mol name	Total score
SRC	2h8h	MOL000354	Isorhamnetin	6.8926
		MOL004609	Areapillin	9.1411
		MOL005573	Genkwanin	5.1959
		MOL008039	Isoarcapillin	5.6894
		MOL008040	Eupalitin	6.178
		MOL008041	Eupatolitin	8.4508
		MOL008046	Demethoxycapillarisin	4.7983
		MOL000098	Quercetin	5.8955
		MOL000853	alisol B	6.197
		MOL000822	Polyporusterone G	3.5684
		MOL000022	14-acetyl-12-senecioyl-2E,8Z,10E-atractylentriol	6.2891
PIK3CA	4jps	MOL002464	1-Monolinolein	7.9419
		MOL000279	Cerevisterol	2.3226
		MOL000796	(22e,24r)-ergosta-6-en-3beta,5alpha,6beta-triol	3.3951
		MOL000820	Polyporusterone E	3.4059
		MOL000830	Alisol B	6.2659
		MOL000831	Alisol B monoacetate	4.2789
		MOL000853	alisol B	4.8636
		MOL000822	polyporusterone G	4.636
		MOL000020	12-senecioyl-2E,8E,10E-atractylentriol	7.3697
		MOL000021	14-acetyl-12-senecioyl-2E,8E,10E-atractylentriol	7.4433
		MOL000022	14-acetyl-12-senecioyl-2E,8Z,10E-atractylentriol	5.6394
CASP3	4ehd	MOL000820	Polyporusterone E	4.2518
		MOL000020	12-senecioyl-2E,8E,10E-atractylentriol	4.8703

**FIGURE 3:**
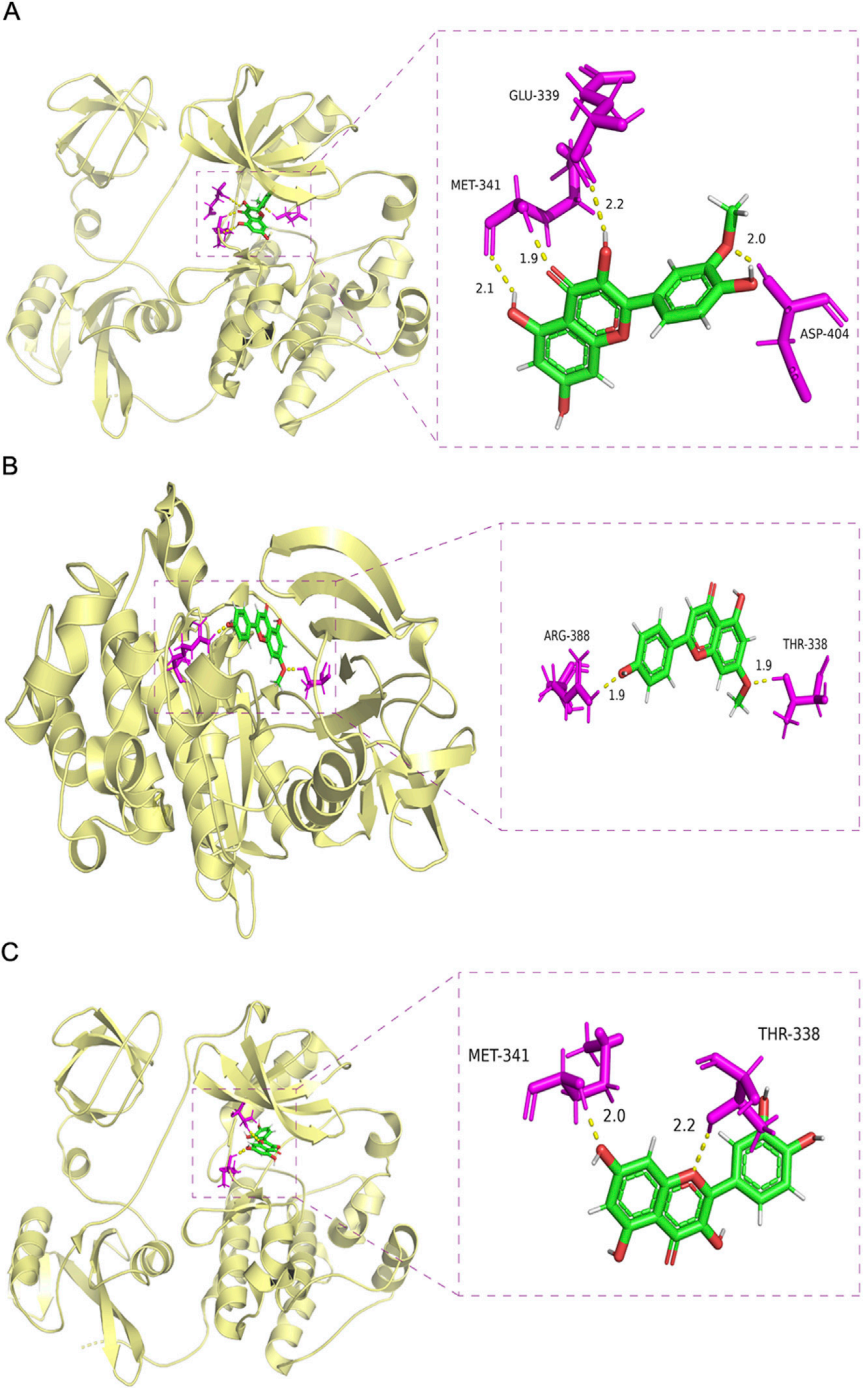
Molecular docking of selected active compounds with the core AML-related target protein SRC. **(A)** Isorhamnetin-SRC. **(B)** Genkwanin-SRC. **(C)** Quercetin-SRC.

To further illustrate these interactions, Figure 3 presents the docking conformations of three representative YWLS compounds—Isorhamnetin, Genkwanin, and Quercetin—with SRC, a non-receptor tyrosine kinase implicated in AML cell proliferation and survival. All three compounds were predicted to stably occupy the active binding pocket of SRC, engaging in a combination of hydrogen bonding and hydrophobic interactions that support high-affinity binding. These interactions may underlie the potential of YWLS components to inhibit SRC-mediated oncogenic signaling, reinforcing their candidacy as therapeutic agents in AML (Lohning et al., 2017).

### ADME prediction analysis

ADME prediction using the SwissADME database assessed the pharmacokinetic properties of selected YWLS compounds. Fifteen active ingredients met key druggability indicators (Table 3), demonstrating favorable gastrointestinal absorption, bioavailability, and druglikeness. Flavonoids like Genkwanin, Isorhamnetin, and Quercetin were particularly promising, while compounds such as Eupalitin could cross the blood-brain barrier, suggesting broader therapeutic potential. These findings support further evaluation of YWLS compounds for AML treatment, including their pharmacokinetic profiles and safety.

**TABLE 3:** Pharmacokinetic properties of selected YWLS compounds by ADME prediction analysis.

ADME feature		Pharmacokinetics				Druglikeness		Medical chemistry	
Mol id	Molecule name	GI absorption	BBB permeant	P-gp substrate	Inhibition of liver drug enzyme (5)	The number of druggability indicators	Bioavailability score	Pains	Synthetic accessibility
MOL000354	Isorhamnetin	High	No	No	3	5	0.55	0	3.26
MOL004609	Areapillin	High	No	No	4	5	0.55	0	3.52
MOL005573	Genkwanin	High	No	No	4	5	0.55	0	3.03
MOL008039	Isoarcapillin	High	No	No	4	5	0.55	1	3.53
MOL008040	Eupalitin	High	Yes	No	4	5	0.55	0	3.4
MOL008041	Eupatolitin	High	No	No	4	5	0.55	1	3.48
MOL008046	Demethoxycapillarisin	High	No	No	3	5	0.55	0	3.36
MOL000098	quercetin	High	No	No	3	5	0.55	1	3.23
MOL000853	alisol B	High	No	No	0	4	0.55	0	6.15
MOL000822	polyporusterone G	High	No	Yes	1	4	0.55	0	6.32
MOL000830	Alisol B	high	No	No	0	4	0.55	0	6.4
MOL000831	Alisol B monoacetate	high	No	No	0	1	0.17	0	6.55
MOL000020	12-senecioyl-2E,8E,10E-atractylentriol	High	Yes	No	2	5	0.85	0	4.36
MOL000021	14-acetyl-12-senecioyl-2E,8E,10E-atractylentriol	High	Yes	No	3	5	0.56	0	4.53
MOL000820	polyporusterone E	High	No	Yes	0	4	0.55	0	6.35
MOL000276	7,9 (11)-dehydropachymic acid	Low	No	Yes	1	1	0.56	0	6.7
Enasidenib	2-methyl-1-[[4-[6-(trifluoromethyl)-2-pyridinyl]-6-[[2-(trifluoromethyl)-4-pyridinyl]amino]-1,3,5-triazin-2-yl]amino]-2-propanol	Low	No	No	4	2	0.55	0	3.18
Giltertinib	6-ethyl-3-[[3-methoxy-4-[4-(4-methyl-1-piperazinyl)-1-piperidinyl]phenyl]amino]-5-[(tetrahydro-2H-pyran-4-yl)amino]-2-pyrazinecarboxamide	High	No	No	1	3	0.17	1	4.73
Glasdegib	1-((2R,4R)-2-(1H-benzo [d]imidazole-2-yl)-1-methylpiperidin-4-yl)-3-(4-cyanophenyl)urea maleate	High	No	Yes	3	5	0.55	0	3.63
Ivosidenib	(2S)-1-(4-cyano-2-pyridinyl)-5-oxo-L-prolyl-2-(2-chlorophenyl)-N-(3,3-difluorocyclobutyl)-N2-(5-fluoro-3-pyridinyl)-glycinamide	Low	No	Yes	4	4	0.55	0	4.42
Midostaurin	N-benzoylstaurosporine	High	No	Yes	4	3	0.55	0	5.41

### Molecular dynamics simulation

**FIGURE 4:**
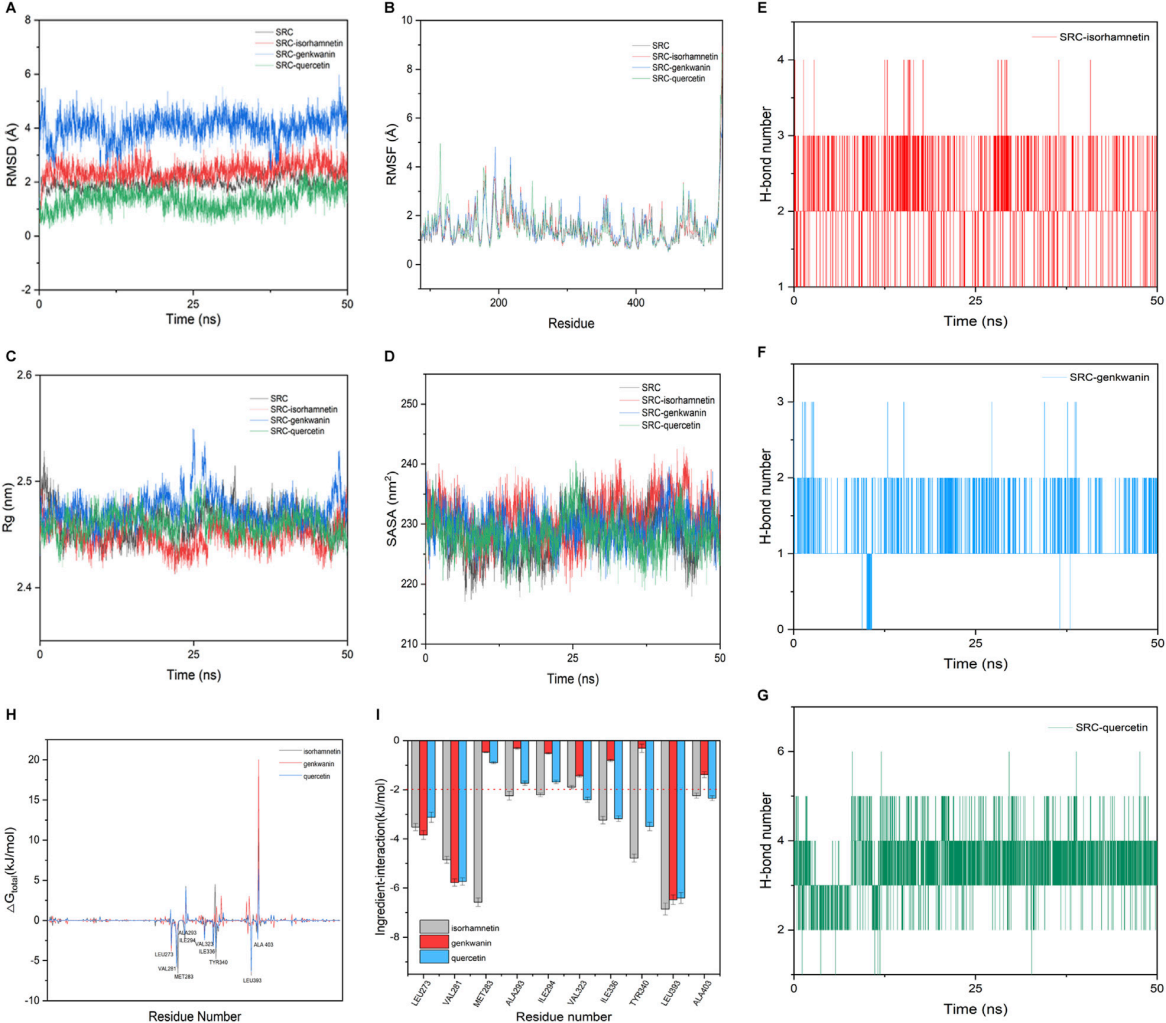
Molecular dynamics simulation of Genkwanin, isorhamnetin, and quercetin and SRC. **(A)** RMSD of Genkwanin, isorhamnetin, and quercetin in complex with SRC, respectively. **(B)** RMSF of amino acid residues of Genkwanin, isorhamnetin, and quercetin in complex with SRC, respectively. **(C)** Rg of Genkwanin, isorhamnetin, and quercetin in complex with SRC, respectively. **(D)** SASA of Genkwanin, isorhamnetin, and quercetin in complex with SRC, respectively. **(E–G)** H-bond number of isorhamnetin, Genkwanin, and quercetin in complex with SRC, respectively. **(H)** Gibbs binding free energy of isorhamnetin, Genkwanin, and quercetin in complex with SRC, respectively. **(I)** Interaction energy between the isorhamnetin, Genkwanin, and quercetin and key amino acid residues of SRC, respectively.

The RMSD values of four systems (unloaded SRC, SRC-isorhamnetin, SRC-genkwanin, and SRC-quercetin) reached equilibrium after a simulation period (Figure 4A). The RMSD values were 1.97 Å, 2.38 Å, 4.07 Å, and 1.34 Å, respectively, indicating that the complexes were stable. As shown in Figure 4B, the RMSF values varied among the systems due to different interactions between the active molecules and SRC. Despite these differences, the RMSF trends were similar, with notable increases in the regions between residues 113–117 and 519–529, where the SRC loop structure exhibits flexible space. Figure 4C shows that the radius of gyration (RG) of unloaded SRC (2.46 nm), SRC-isorhamnetin (2.45 nm), SRC-genkwanin (2.47 nm), and SRC-quercetin (2.46 nm) remained stable during the simulation, confirming that the complex structures remained stable throughout binding, consistent with the RMSD analysis.

Figure 4D shows that the overall solvent accessible surface area (SASA) of these complexes showed minimal changes from the unloaded SRC, suggesting that the binding of isorhamnetin, genkwanin, and quercetin had little effect on the hydrophilicity and hydrophobicity of SRC. The role of hydrogen bonds in maintaining the stability of these complexes is shown in Figures 4E–G. All three compounds formed hydrogen bonds with SRC, with quercetin forming the most frequent bonds, followed by isorhamnetin and genkwanin, highlighting quercetin’s significant role in binding to SRC.

To calculate the binding free energy, we selected the RMSD stationary phase in the last 5 ns of the simulation and extracted one conformation every 10 ps, resulting in 500 conformations. The binding free energies and components for the SRC-isorhamnetin, SRC-genkwanin, and SRC-quercetin complexes are presented in Table 4. The binding free energies were −60.42 ± 1.48 kJ/mol for SRC-isorhamnetin, −48.78 ± 1.59 kJ/mol for SRC-genkwanin, and −61.04 ± 1.49 kJ/mol for SRC-quercetin. The van der Waals energies were −148.66 ± 1.86 kJ/mol for SRC-isorhamnetin, −117.71 ± 1.36 kJ/mol for SRC-genkwanin, and −132.77 ± 1.72 kJ/mol for SRC-quercetin. The electrostatic energies were −70.20 ± 1.07 kJ/mol, −44.96 ± 0.91 kJ/mol, and −77.18 ± 1.64 kJ/mol, respectively. Additionally, the polar solvation energies were 175.18 ± 1.40 kJ/mol, 129.92 ± 0.92 kJ/mol, and 164.73 ± 1.06 kJ/mol. These findings indicate that van der Waals and electrostatic interactions significantly contribute to binding, while polar solvation inhibits interaction.

**TABLE 4:** Binding free energy and energy components of (isorhamnetin, genkwanin, quercetin)-SRC complex.

Complex	△Evdw (kJ/mol)	△Eele (kJ/mol)	△Gpolar (kJ/mol)	△Gsasa (kJ/mol)	△Gbinding (kJ/mol)
Isorhamnetin-SRC	−148.657 ± 1.856	−70.201 ± 1.073	175.180 ± 1.403	−16.728 ± 0.096	−60.416 ± 1.480
Genkwanin-SRC	−117.712 ± 1.364	−44.960 ± 0.906	129.922 ± 0.921	−16.029 ± 0.109	−48.781 ± 1.587
Quercetin-SRC	−132.766 ± 1.721	−77.178 ± 1.638	164.726 ± 1.061	−15.835 ± 0.110	−61.041 ± 1.489

To further investigate the binding mechanism, we used residue-free energy decomposition to identify key residues contributing to the binding of isorhamnetin, genkwanin, and quercetin to SRC. Figure 4H compares the binding free energy of each compound with SRC, while Figure 4I highlights the key residues contributing to the binding process. The residues LEU273, VAL281, VAL323, LEU393, and ALA403 were identified as significant contributors to binding across all three complexes. The binding energies of these residues were as follows: −3.5, −4.85, −1.90, −6.86, −2.24 kJ/mol for isorhamnetin; −3.84, −5.78, −1.44, −6.48, −1.39 kJ/mol for genkwanin; and −3.84, −5.78, −1.44, −6.48, −1.39 kJ/mol for quercetin. Additionally, in the isorhamnetin-SRC complex, nine SRC residues (LEU273, VAL281, MET283, ALA293, ILE294, ILE336, TYR340, LEU393, and ALA403) interacted with isorhamnetin with binding energies greater than −2 kJ/mol.

### Cytotoxic effects of Quercetin, Isorhamnetin, and Genkwanin on AML cells

**FIGURE 5:**
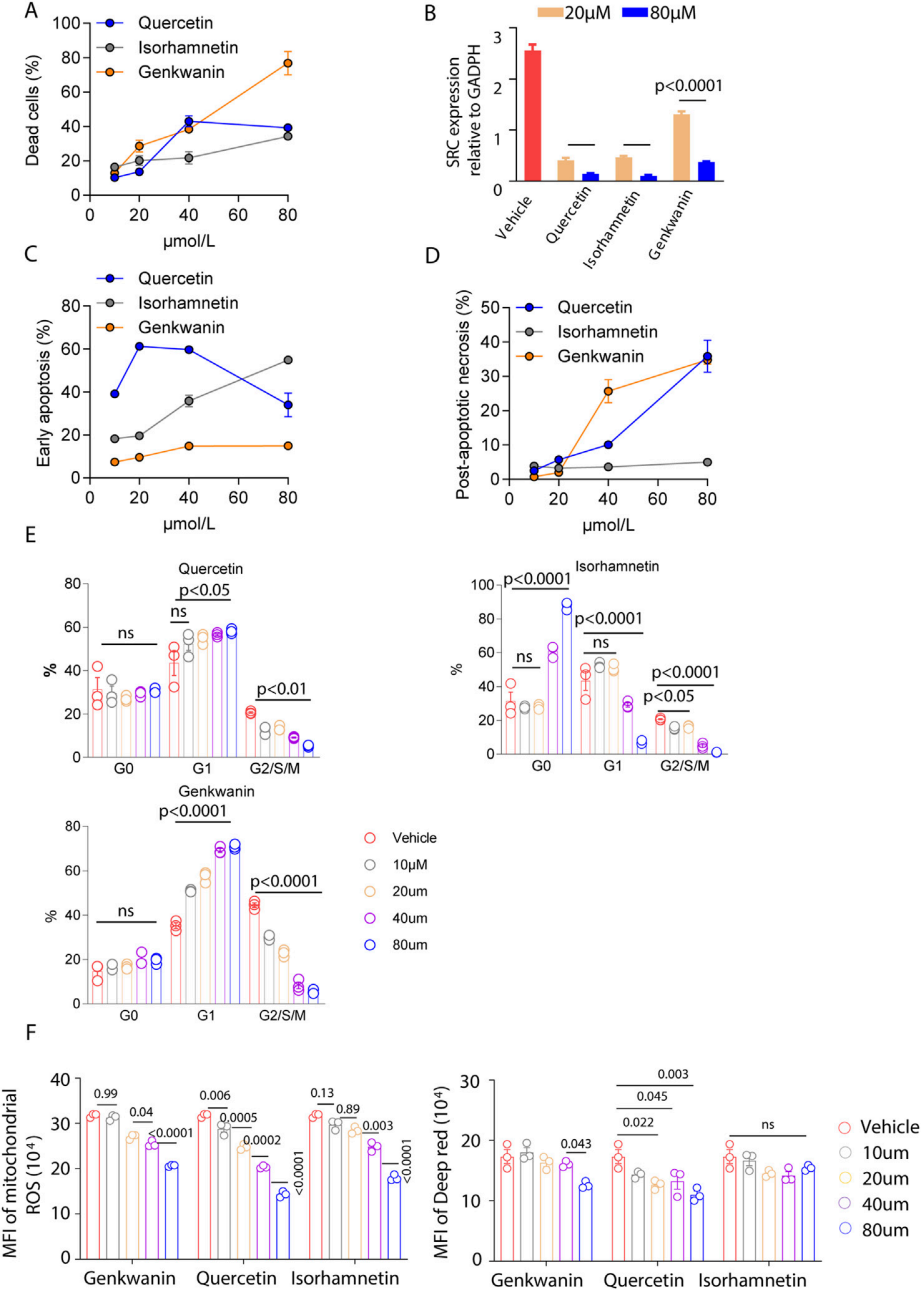
Effects of Genkwanin, Isorhamnetin, and Quercetin on leukemia cell apoptosis, cell cycle, and mitochondrial function. **(A)** Cytotoxic effects of Genkwanin, Isorhamnetin, and Quercetin on leukemic cells, presented relative to the vehicle control. **(B)** SRC expression in leukemic cells at 72 h following treatment with different concentrations of Genkwanin, Isorhamnetin, and Quercetin. **(C)** Early apoptosis of leukemic cells at 72 h after treatment with different concentrations of Genkwanin, Isorhamnetin, and Quercetin. **(D)** Late apoptosis of leukemic cells at 72 h after treatment with different concentrations of Genkwanin, Isorhamnetin, and Quercetin. **(E)** Cell cycle distribution of leukemic cells at 72 h after treatment with different concentrations of Genkwanin, Isorhamnetin, and Quercetin. **(F)** Reactive oxygen species levels and mitochondrial membrane potential in leukemic cells at 72 h following treatment with different concentrations of Genkwanin, Isorhamnetin, and Quercetin.

To assess the cytotoxic effects of selected YWLS compounds on AML cells, MOLM14, a human acute myeloid leukemia cell line, was treated with varying concentrations of Quercetin, Isorhamnetin, and Genkwanin for 72 h. Cell viability was evaluated using eFluor™ 506 Viability Dye staining followed by flow cytometry. The results demonstrated a clear dose-dependent reduction in cell viability for all three compounds, compared to the vehicle-treated control. Among them, Genkwanin exhibited the strongest cytotoxic effect, followed by Isorhamnetin and Quercetin, suggesting that these compounds may exert anti-leukemic activity through direct effects on AML cell survival (Figure 5A).

Based on the fitted dose-response models, predicted inhibition rates at 0.1, 1, 10, and 100 μM were obtained for each compound. IC50 analysis for Quercetin, Isorhamnetin, and Genkwanin derived from four-parameter logistic curve fitting were as follows: Quercetin: 24.49 μM, Isorhamnetin: 162.41 μM, and Genkwanin: 55.71 μM. As shown in Supplementary Table S5, inhibition was minimal at lower concentrations (≤10 μM), consistent with experimental observations in Figure 5A. Genkwanin exhibited a marked increase in predicted inhibition at 100 μM, consistent with its steep dose-response profile.

Since previous analyses suggested that these compounds target SRC, qRT-PCR was performed to evaluate SRC expression in MOLM14 cells post-treatment. A significant downregulation of SRC was observed at concentrations of 20 µM and 80 μM, compared to the vehicle group (Figure 5B), indicating that SRC is a viable target for these compounds. These findings suggest that these compounds may be promising pharmacological agents for AML treatment.

To further investigate the mechanisms underlying the observed cytotoxic effects, we performed an apoptosis assay using Annexin V staining. Isorhamnetin treatment induced early apoptosis in MOLM14 cells in a dose-dependent manner, with lower concentrations of Quercetin also promoting early apoptosis (Figure 5C; Supplementary Figure S4). Genkwanin demonstrated a mild enhancement of early apoptosis. For late apoptosis (Figure 5D; Supplementary Figure S4), both Quercetin and Genkwanin significantly increased post-apoptotic necrosis, whereas Isorhamnetin had a minimal effect. Overall, these compounds appear to induce tumor cell death primarily by promoting apoptosis.

Additionally, cell cycle analysis revealed distinct effects of the compounds on the cell cycle. Quercetin promoted G1/S/M phase arrest, even at low concentrations (Figure 5E). High concentrations of Isorhamnetin caused an increase in the G0 phase, keeping tumor cells in a quiescent state while reducing progression through the G1/S/M phases. Genkwanin exhibited a dose-dependent increase in the G1 phase and G2/S/M phase arrest, with these effects being more pronounced at higher concentrations.

Mitochondrial function is critical for tumor cell proliferation, as mitochondria are central to cellular energy metabolism and survival. Previous studies have emphasized the importance of mitochondrial alterations in cancer progression and immunity (Li et al., 2024; Wang et al., 2023). In our study, we assessed mitochondrial reactive oxygen species (ROS) levels and membrane potential, finding that all three compounds significantly reduced ROS levels and mitochondrial membrane potential (Figure 5F). These results suggest that the compounds may disrupt the cellular energy balance, impairing leukemia cell metabolism and inhibiting cell proliferation.

In conclusion, Quercetin, Isorhamnetin, and Genkwanin exhibit cytotoxic effects against AML cells through the induction of apoptosis, modulation of the cell cycle, and disruption of mitochondrial function, highlighting their potential as therapeutic agents.

### Genkwanin as a therapeutic pharmacological target for AML

The three compounds demonstrated significant cytotoxicity against AML cells, prompting us to investigate their potential impact on immune cells, which play a critical role in modulating the tumor microenvironment. A T cell proliferation assay revealed that quercetin and genkwanin did not significantly affect CD4^+^ T cell proliferation, as measured by CFSE, compared to the vehicle group (Supplementary Figure S3A). However, isorhamnetin exhibited a dose-independent proliferative effect. For CD8^+^ T cells, quercetin and isorhamnetin had minimal effects on proliferation, whereas genkwanin showed a pronounced proliferative effect at 10 μM, comparable to effects observed at higher concentrations (20 μM and 80 µM) (Supplementary Figure S3B). These *in vitro* findings suggest that the tested compounds are not toxic to healthy splenic T cells and may modulate T cell activity. Given the importance of CD8^+^ T cells in anti-leukemia immunity, these observations provided the rationale for subsequent *in vivo* studies to evaluate whether genkwanin could enhance anti-leukemia immune responses within the tumor microenvironment.

**FIGURE 6:**
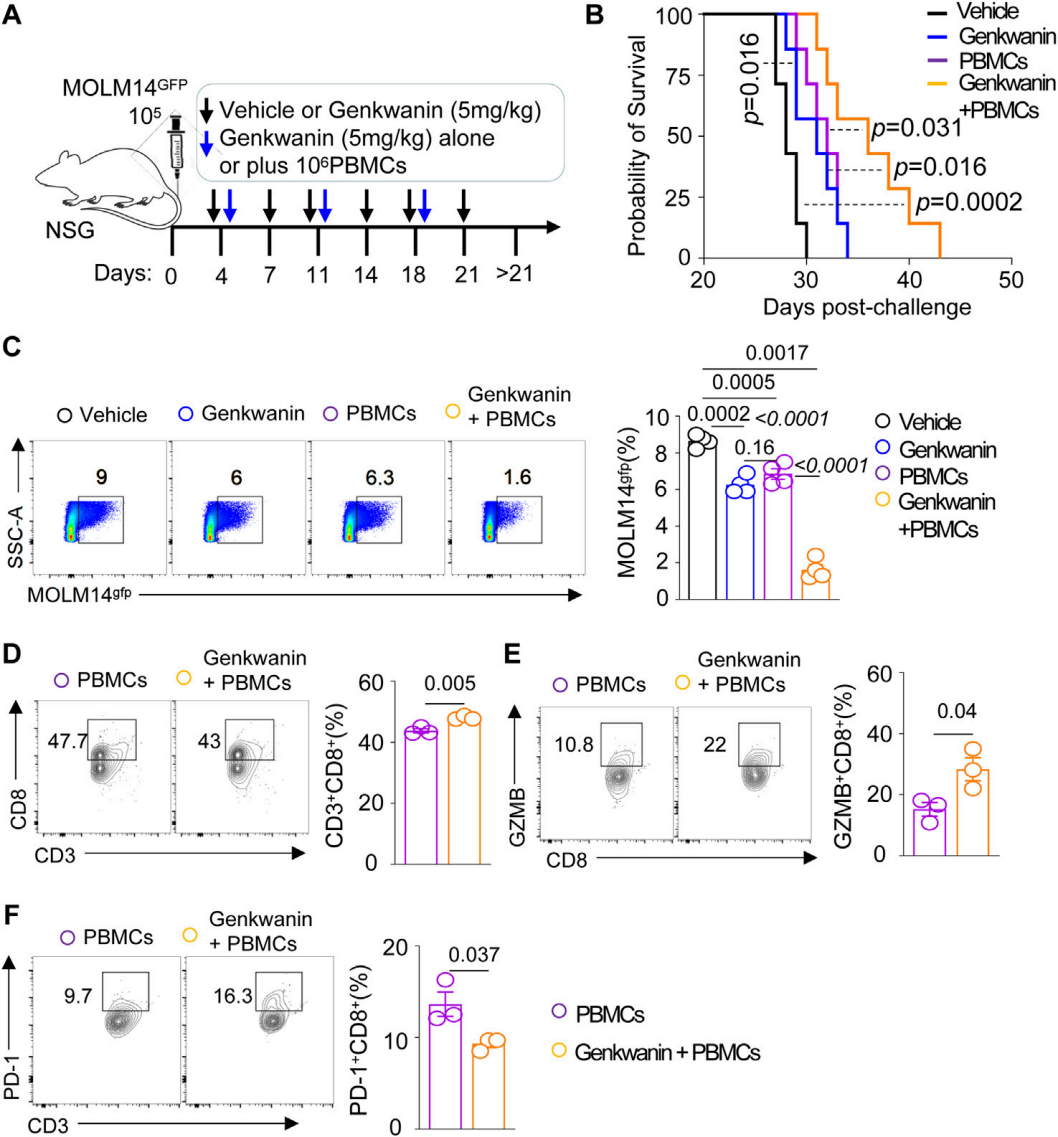
Genkwanin as a pharmacological agent in a leukemia model. **(A)** Schematic representation of Genkwanin treatment alone or in combination with adoptive PBMC transfer. **(B)** Kaplan-Meier survival curve of mice treated with Genkwanin alone or in combination with adoptive PBMC transfer. **(C)** Frequency of leukemia cells in the bone marrow on Day 21 post-challenge. **(D)** Frequency of CD3^+^CD8^+^T cells in the bone marrow in mice transferred with PBMCs alone or Genkwanin + PBMCs on Day 21 post-challenge. **(E)** Frequency of GZMB^+^CD8^+^T cells in the bone marrow in mice transferred with PBMCs alone or Genkwanin + PBMCs on Day 21 post-challenge. **(F)** Frequency of PD-1^+^CD8^+^T cells in the bone marrow in mice transferred with PBMCs alone or Genkwanin + PBMCs on Day 21 post-challenge.

We utilized an established leukemia xenograft model by injecting 1 × 10^5^ MOLM14gfp cells into NSG mice (Figure 6A). Genkwanin was administered intraperitoneally at 5 mg/kg starting on day 4 post-leukemia cell injection, with a total of six doses given twice weekly. Additional treatment groups included mice receiving genkwanin in combination with weekly adoptive transfer of 1 × 10^6^ human PBMCs, as well as mice treated with PBMCs alone. The results showed that genkwanin monotherapy significantly prolonged survival compared to the vehicle-treated group (Figure 6B). However, there was no significant difference between the genkwanin-only group and the PBMC-only group. Notably, mice treated with the combination of genkwanin and PBMCs exhibited further improved survival and a marked reduction in leukemia burden by Day 21 post-challenge, compared to genkwanin treatment alone (Figure 6C).

To determine whether genkwanin modulates human T cell responses *in vivo*, we analyzed the bone marrow of NSG mice co-engrafted with MOLM14 cells and human PBMCs. Flow cytometric analysis revealed an increased frequency of human CD3^+^CD8^+^ T cells in mice receiving genkwanin plus PBMCs compared to those treated with PBMCs alone (Figure 6D). A similar trend was observed for granzyme B (GZMB)^+^CD8^+^ T cells, suggesting enhanced cytotoxic potential (Figure 6E). Moreover, the frequency of PD-1^+^CD8^+^ T cells was reduced in the genkwanin-treated group (Figure 6F), indicating a possible alleviation of T cell exhaustion. Collectively, these data suggest that genkwanin may promote the expansion and functional activation of CD8^+^ T cells *in vivo*, and may hold promise as a potential pharmacological target for AML therapy.

## Discussion

AML is a highly aggressive and prevalent hematological malignancies, characterized by the accumulation of immature myeloid progenitor in the bone marrow, resulting in impaired hematopoiesis and poor clinical outcomes (Garcia-Manero, 2023; Fu et al., 2023; Huang et al., 2019). Despite advancements in targeted therapies—such as FLT3, IDH, and BCL-2 inhibitors—treatment resistance and relapse rates remain high, and the 5-year survival rate for AML patients remains disappointingly low (Zhang B. et al., 2021; Forsberg and Konopleva, 2024; Mohamed Jiffry et al., 2023). Immunotherapies, including monoclonal antibodies (e.g., gemtuzumab ozogamicin targeting CD33), bispecific T-cell engagers (BiTEs), and CAR T-cell therapies targeting antigens like CD123, represent promising modalities but are limited by antigen heterogeneity, toxicity, off-target effects, and the immuosuppressive bone marrow microenvironment (Restelli et al., 2024; Abaza et al., 2024).

In this context, traditional Chinese medicine (TCM) offers a holistic approach, focusing on restoring physiological balance and supporting immune function (Xiang et al., 2019; Zhang et al., 2024; Kim et al., 2024; Yang et al., 2020). In the context of AML, specific herbal compounds such as curcumin, berberine, and astragalus polysaccharides exhibit anti-inflammatory, antioxidant, and pro-apoptotic effects, targeting critical pathways involved in disease progression, including apoptosis, angiogenesis, and metastasis (de Waure et al., 2023; Huang et al., 2023; Yang et al., 2024). In addition to these direct anti-leukemic agents, broader traditional Chinese medicine (TCM) therapies have been employed to alleviate treatment-related side effects—such as fatigue, nausea, and immune suppression—commonly experienced by AML patients undergoing chemotherapy or stem cell transplantation (Ling, 2013). The integration of TCM with contemporary AML treatments holds considerable promise. For instance, TCM may complement targeted therapies like FLT3 and IDH inhibitors or immune-based approaches, such as CAR T-cell therapy, by enhancing immune responses, mitigating side effects, and potentially improving therapeutic outcomes (Ruglioni et al., 2024; Ge et al., 2022; Marvin-Peek et al., 2022). Ongoing research and clinical trials are actively exploring the synergistic effects of combining TCM with chemotherapy, targeted therapies, and immunotherapies, aiming to offer more effective and holistic treatment strategies for AML patients (Liu et al., 2020; Wu et al., 2024; Shi et al., 2021).

TCM bridges the gap between traditional and modern approaches, offering a promising avenue for integrated cancer care in AML. YWLS is believed to regulate Qi and improve blood flow, alleviating cancer-related fatigue and improving patients’ overall wellbeing (Su and Tai, 2020). Studies suggest that its anti-inflammatory properties play a key role in inhibiting tumor progression and metastasis (Mou et al., 2022). Additionally, its detoxifying and liver-protecting effects may reduce liver damage caused by cancer treatments, enhancing chemotherapy efficacy (Zhu et al., 2016). Flavonoids in YWLS, known for their anti-cancer properties, inhibit tumor proliferation and induce apoptosis by downregulating the PI3K/AKT/mTOR signaling pathway (Kopustinskiene et al., 2020; Mir et al., 2024; Zhang et al., 2018). Given the complex composition of TCM formulations, preclinical studies have focused on identifying the molecular targets and mechanisms of individual components within YWLS. For example, Genkwanin—a flavonoid present in several medicinal plants—has demonstrated potential anticancer activity, particularly in head and neck squamous cell carcinoma, and emerging evidence suggests that Genkwanin may exert its effects by inducing apoptosis and inhibiting cancer cell proliferation (Grattan, 2013; Zhang B. et al., 2022). While these findings are encouraging, they relate to specific active compounds rather than the entire YWLS formulation. Therefore, further investigation into the individual constituents of YWLS and their role in tumorigenesis is warranted. Such studies could provide a scientific foundation for the potential integration of these herbal components into broader cancer treatment strategies.

Our study highlights the potential of YWLS, a classical TCM formula, as a novel source of anti-leukemia agents. In this study, we identified 58 active ingredients of YWLS from the TCMSP database. Some ingredients have been implicated in tumorigenesis, suggesting their potential role in treating leukemia. Among these, cerevisterol and (22E, 24R)-ergosta-6-en-3β, 5α, 6β-triol are steroids known for their anti-inflammatory, immunomodulatory, and anti-cancer activities (Calpe-Berdiel et al., 2007). Steroids have shown efficacy in cancers such as breast (Grattan, 2013), gastric (De Stefani et al., 2000), and lung (Mendilaharsu et al., 1998). Polyporusterone E has a dose-independent inhibitory effect in the cell proliferation of leukemia L-1210 (Ohsawa et al., 1992). Additionally, Genkwanin, a major non-glycosylated flavonoid, exhibits anti-inflammatory effects by modulating the miR-101/MKP-1/MAPK pathway (Gao et al., 2014). Isorhamnetin has demonstrated anti-tumor activity across cancers, including colorectal, skin, lung, and breast, through apoptosis induction and signaling pathway inhibition (Ruan et al., 2015; Hu et al., 2015). A total of 113 target genes were identified for YWLS active ingredients, intersecting with AML-related genes. Pathway analysis revealed enrichment in PI3K-AKT, MAPK, JAK-STAT, PD-1/PD-L1, and leukemia-specific signaling pathways, which are closely linked to AML carcinogenesis and drug resistance (Park et al., 2010). Among these, 10 genes correlated with favorable survival (e.g., *PIK3CA/B*, *ITGA4*, *CCND2*, *MPO*, and *CASP3*), while others, such as *PIM1* (Cheng et al., 2017), *IDH1* (Messina et al., 2022), *SRC* (Patel et al., 2019), and *CCND3* (Matsuo et al., 2018), were associated with poor survival, consistent with previous evidence. High *CCND3* and *SRC* expression, validated in an independent dataset, correlated with unfavorable survival. Dysregulated D-type cyclins (e.g., *CCND1*, *CCND2*, and *CCND3*) drive the G1-to-S phase cell cycle transition via CDK4/6, contributing to AML pathogenesis (Matsuo et al., 2018; Choi et al., 2012). Src, a tyrosine kinase, regulates cell proliferation, motility, and survival and is implicated in AML drug resistance (Ozawa et al., 2008). While inhibitors like A-419259 and TL02-59 target Src family kinases such as Hck and Fgr, acquired resistance remains a significant challenge (Yang et al., 2022; Negi et al., 2021), underscoring the need for novel inhibitors to improve AML outcomes.

Using molecular docking, we identified several compounds from the active ingredients of YWLS that are predicted to target SRC, including quercetin, isorhamnetin, alisol B, eupalitin, eupatolitin, areapillin, and genkwanin. To evaluate the drug potential of these compounds for AML patients, ADME analysis revealed that most SRC-targeting compounds possess favorable pharmacological properties, including good drug-likeness and bioavailability scores comparable to current clinical agents. Flavonoids such as genkwanin, isorhamnetin, and quercetin exhibited excellent gastrointestinal absorption, druggability, and bioavailability. Notably, eupalitin, 12-senecioyl-2E, 8E, 10E-atractylentriol, and 14-acetyl-12-senecioyl-2E, 8E, 10E-atractylentriol demonstrated blood-brain barrier penetration but may accumulate in the body due to inhibition of liver drug-metabolizing enzymes. Flavonoids are known to suppress cancer cell growth by inhibiting proliferation and inducing apoptosis through the downregulation of the PI3K/AKT/mTOR/ULK signaling pathway (Mir et al., 2024; Zhang et al., 2018; Slika et al., 2022). In *in vitro* killing assays, the compounds inhibited AML cell growth to varying degrees and reduced SRC expression in a dose-dependent manner, suggesting their potential as therapeutic agents for AML.

We further focused on quercetin, isorhamnetin, and genkwanin due to their commercial availability and supporting evidence from previous studies. Molecular dynamics simulations indicated that the binding stability of quercetin and isorhamnetin to the SRC protein skeleton was greater than that of genkwanin. Despite this, the flexibility and intensity of protein-ligand movements during binding were comparable across the three compounds. Hydrogen bonding played a crucial role in stabilizing the interactions between SRC and these compounds, as reported in prior studies (Huang et al., 2014; Chen et al., 2016). The binding stability correlated with the comparable number of hydrogen bonds formed. Free energy analysis identified key amino acid residues critical to binding stability (Chen J. et al., 2024). For example, MET283 and ILE336 were crucial for isorhamnetin binding, while LEU273 and LEU393 were essential for all three compounds. These findings provide a molecular-level foundation for targeting SRC with these compounds. However, further validation through site-directed mutagenesis is necessary to confirm these results (Liao et al., 2001).

In parallel, dose-response modeling using the four-parameter logistic (4 PL) model provided valuable insights into the tumor cell growth inhibition rates of Quercetin, Isorhamnetin, and Genkwanin at various concentrations. The model predicted minimal inhibition at concentrations ≤10 μM, consistent with experimental observations, indicating that higher concentrations are needed for significant anti-tumor effects. Genkwanin showed a notably steep dose-response curve, as evidenced by the sharp increase in inhibition at 100 μM. This behavior highlights its potential for greater efficacy at higher doses, supporting its consideration for further investigation in therapeutic applications. In contrast, Quercetin and Isorhamnetin showed more gradual increases in tumor growth inhibition, achieving approximately 40% inhibition at 100 μM. These findings suggest that while Quercetin and Isorhamnetin may have moderate anti-tumor effects, their potency may be limited at concentrations typically used in clinical or experimental settings.

Beyond tumor cell inhibition, CD8^+^ T cells are central to anti-tumor immunity, but their exhaustion often renders them incapable of effective tumor killing (Desai et al., 2023). Among the three compounds, genkwanin demonstrated a superior ability to promote T-cell proliferation, particularly of CD8^+^ T cells, even at low concentrations. Genkwanin also reduced mitochondrial ROS levels, suggesting it may restore T-cell function while inhibiting tumor growth. These observations align with previous studies showing genkwanin induces apoptosis, mitigates oxidative stress, and scavenges free radicals (El Menyiy et al., 2023). In addition, genkwanin induced G2/S/M cell cycle arrest in AML cells. In a leukemia xenograft model, genkwanin significantly inhibited leukemic cell growth, improved survival, and did not cause observable toxicity, such as colitis. These data suggest that genkwanin’s dual-targeting strategy—enhancing CD8^+^ T-cell function and inhibiting leukemia cell growth—holds promise as a therapeutic strategy for AML by SRC targeting.

Compared to current targeted therapies, which often address single genetic mutations (e.g., FLT3, IDH1/2), the multi-targeted nature of YWLS-derived compounds offers broader anti-leukemia effects and may overcome the limitation of clonal heterogeneity. Additionally, unlike antibody-drug conjugates or CAR-T cells that carry risks of severe cytokine storms, YWLS compounds demonstrated a safer profile with immunomodulatory benefits. These findings suggest that YWLS active ingredients could be developed as either standalone therapies or adjuncts to existing regimens.

Despite these promising results, our study has several limitations that warrant further investigation. One key limitation lies in the target identification process. The use of public databases such as TCMSP and SwissTargetPrediction, although valuable for hypothesis generation, is inherently biased toward well-studied proteins and targets. As a result, potential interactions with less-characterized or novel targets may be underrepresented. This limitation may affect the comprehensiveness of the predicted compound–target interactions and should be considered when interpreting the results. Further studies using unbiased proteomic and transcriptomic approaches are needed to comprehensively map compound-target interactions. The precise mechanism by which these compounds modulate SRC signaling and ROS levels warrants deeper exploration, potentially involving site-directed mutagenesis and metabolic flux analysis. Then, while downregulation of SRC was observed, the precise mechanism remains unclear. Given the complexity of the SRC family, identifying the specific family member targeted by these compounds in AML progression could provide deeper molecular insights. Moreover, combination strategies integrating YWLS ingredients with standard chemotherapies, targeted agents (e.g., FLT3 inhibitors), or immunotherapies (e.g., checkpoint blockade) should be investigated to evaluate potential synergistic effects. Finally, clinical trials assessing the safety, pharmacokinetics, and efficacy of YWLS-derived compounds in AML patients are critical next steps toward translation. In addition, we note that a viability dye was not used prior to mitochondrial staining, which could represent a limitation. Future studies incorporating viability-based gating will further refine analysis of mitochondrial function specifically in surviving cells. Finally, the effect of these compounds on T-cell activation needs to be clarified to strengthen the case for a dual-targeting strategy. Addressing these aspects in future studies will provide a more comprehensive understanding of the therapeutic potential of genkwanin and related compounds in AML.

## Conclusion

In summary, through network pharmacology analysis, molecular docking, and molecular dynamics simulations, we predicted and identified potential therapeutic targets of YWLS active ingredients in the treatment of AML. Three key compounds—genkwanin, quercetin, and isorhamnetin—were validated for their ability to inhibit AML cell growth *in vitro*, with genkwanin demonstrating additional benefits in promoting T-cell proliferation and enhancing survival. These findings highlight the potential of YWLS active ingredients, particularly flavonoids targeting SRC, as promising pharmacological candidates for AML therapy.

Future research should focus on several key areas: (1) further mechanistic exploration to elucidate how individual YWLS compounds modulate SRC signaling, mitochondrial metabolism, and T-cell function; (2) validation of the predicted compound-target interactions using genetic and proteomic approaches; (3) evaluation of combination strategies integrating YWLS compounds with conventional chemotherapies or immunotherapies to assess potential synergistic effects; and (4) advancement toward clinical translation through pharmacokinetic studies, toxicity assessments, and early-phase clinical trials. These efforts will be crucial to fully realize the therapeutic potential of YWLS and its active components in AML treatment.

## Data Availability

The data analyzed in this study are available in the following repositories: 1. TCGA: https://portal.gdc.cancer.gov/; 2. UCSC xena: https://xena.ucsc.edu/; 3. TCMSP: https://old.tcmsp-e.com/tcmsp.php; 4. DisGeNET: https://www.disgenet.org/; 5. GeneCards: https://www.genecards.org/; 6. STRING: https://string-db.org/; 7. RCSB PDB: https://www.rcsb.org; 8. PubChem: https://pubchem.ncbi.nlm.nih.gov/; 9. SwissTargetPrediction: http://www.swisstargetprediction.ch/; 10. SwissADME: http://www.swissadme.ch/.

## Supplementary material

SUPPLEMENTARY FIGURE S1 Workflow of the study.

SUPPLEMENTARY FIGURE S2 GO and KEGG enrichment analysis of intersecting targets. **(A)** The top 10 enriched GO items of BP, CC, and MF. **(B)** The top 20 enriched KEGG pathways. **(C)** The important target genes were mainly distributed in the MAPK signaling pathway. **(D)** The important target genes were mainly distributed in the PI3K-AKT pathway.

SUPPLEMENTARY FIGURE S3 Effects of Genkwanin, Isorhamnetin, and Quercetin on T cells proliferation. **(A)** Effects of Genkwanin, Isorhamnetin, and Quercetin on CD4^+^T cells proliferation. **(B)** Effects of Genkwanin, Isorhamnetin, and Quercetin on murine CD8^+^T cell proliferation.

SUPPLEMENTARY FIGURE S4 Representative flow cytometry plots showing apoptosis induced by Genkwanin, Isorhamnetin, and Quercetin in MOLM14 cells.

SUPPLEMENTARY TABLE S1 Potential targets of active ingredients in YWLS.

SUPPLEMENTARY TABLE S2 Acute myeloid leukemia-related genes from DisGeNET and GeneCards databases.

SUPPLEMENTARY TABLE S3 113 targets were identified from 621 targets of 58 active ingredients in YWLS overlapped with 1,247 genes associated with AML.

SUPPLEMENTARY TABLE S4 Degree value of 20 top targets of YWLS active components analyzed by network analysis.

SUPPLEMENTARY TABLE S5 Predicted tumor growth inhibition (%) at selected concentrations based on model fitting.

## Abbreviations

AML, acute myeloid leukemia; AMK, Atractylodes Macrocephala Koidz; ASH, Artemisiae Scopariae Herba; ADME, absorption, distribution, metabolism, and excretion; AO, Alisma Orientale; BP, Biological Process; CC, Cellular Component; CR, Cinnamomi Ramulus; KEGG, Kyoto Encyclopedia of Genes and Genomes; MCODE. Molecular Complex Detection; MF, Molecular Function; OS, Overall Survival; PU, Polyporus Umbellatus; PPI, Protein-Protein Interaction; PC, Poria Cocos; TCGA, The Cancer Genome Atlas; TCMSP, Traditional Chinese Medicine Systems Pharmacology Database and Analysis Platform; YWLS, Yinchen wuling san.
