# Supplementary figures and images for: Network-based analysis and experimental validation of identified natural compounds from Yinchen Wuling San for acute myeloid leukemia
Source: Front Pharmacol. 2025 May 30;16:1591164. doi: 10.3389/fphar.2025.1591164 (PMC12162526; doi:10.3389/fphar.2025.1591164)

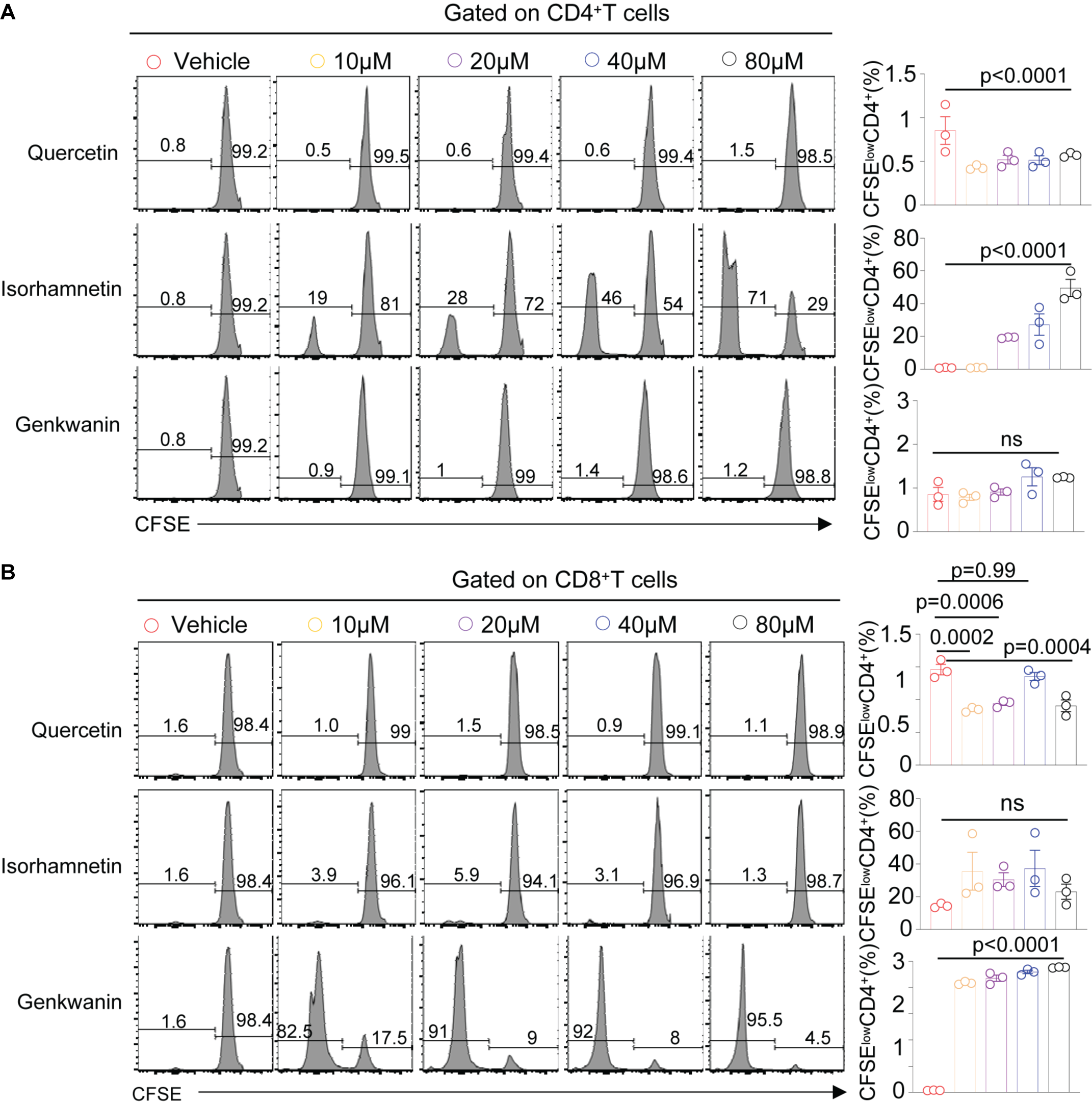

Supplement: Supplementary file 3 [file Image3.tif]

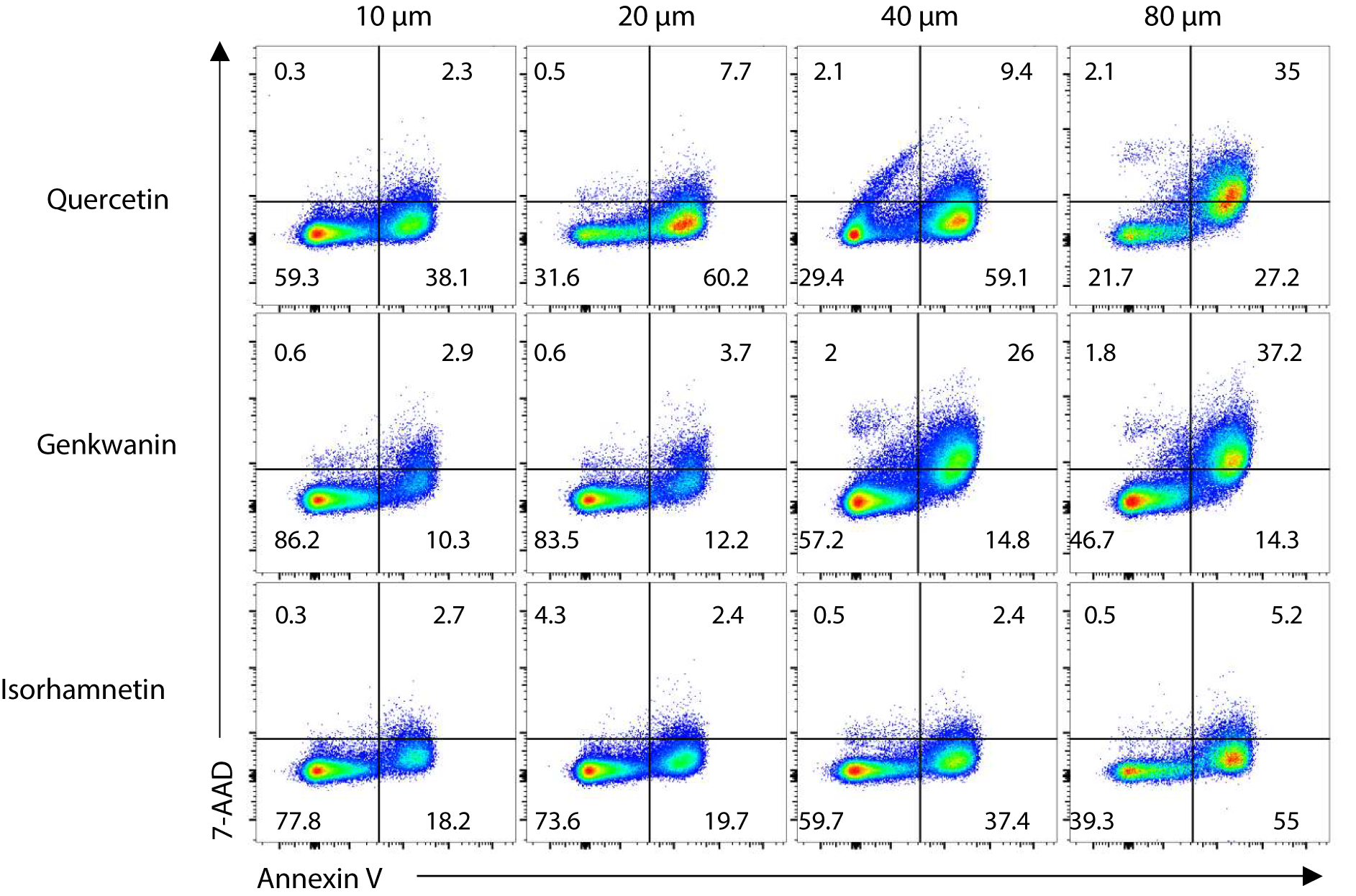

Supplement: Supplementary file 4 [file Image4.tif]

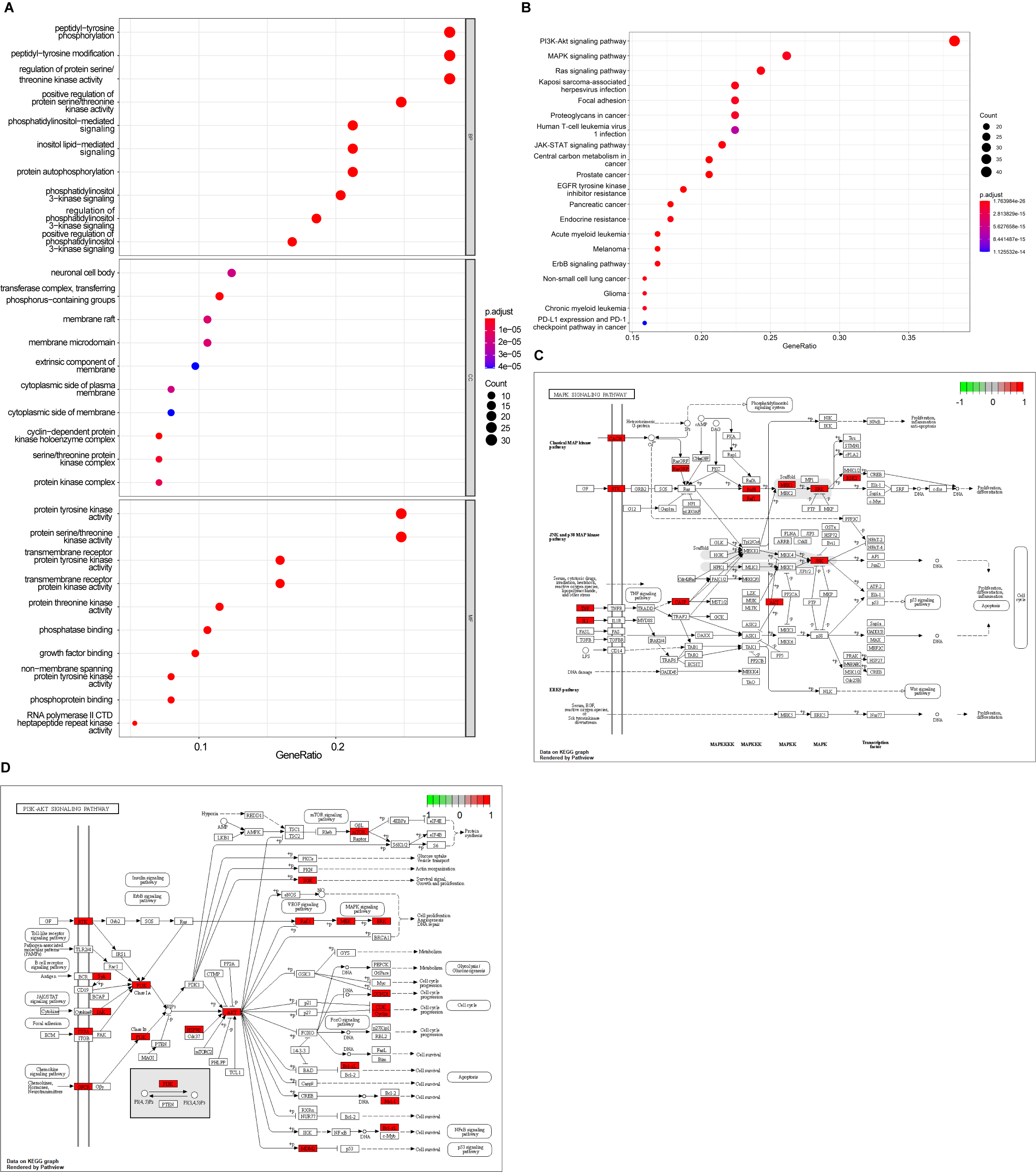

Supplement: Supplementary file 5 [file Image2.tif]

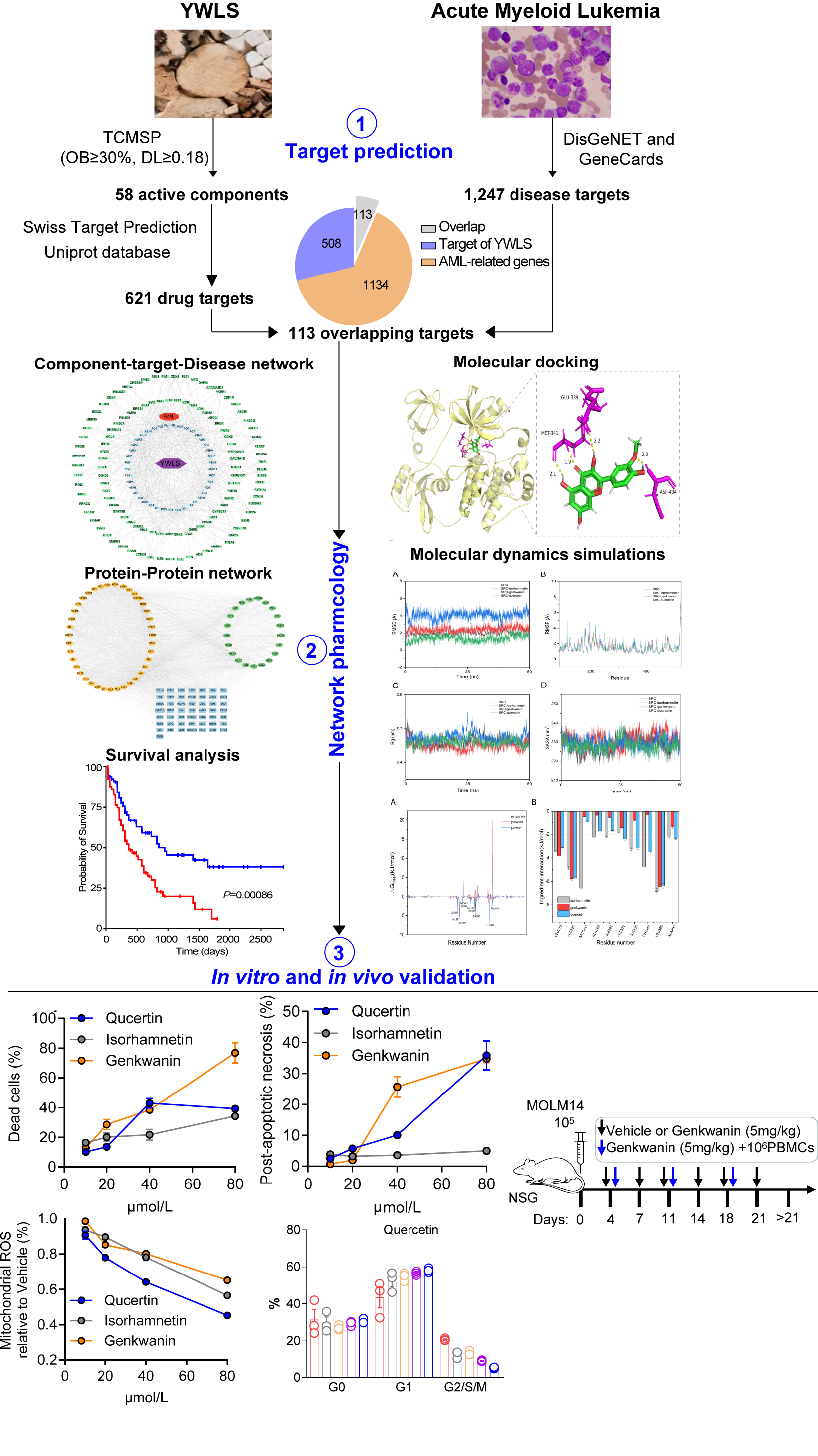

Supplement: Supplementary file 6 [file Image1.tif]
